# KRAS^G12D^ drives immunosuppression in lung adenocarcinoma through paracrine signaling

**DOI:** 10.1172/jci.insight.182228

**Published:** 2025-01-09

**Authors:** Emily L. Lasse-Opsahl, Ivana Barravecchia, Elyse McLintock, Jennifer M. Lee, Sarah F. Ferris, Carlos E. Espinoza, Rachael Hinshaw, Sophia Cavanaugh, Marzia Robotti, Lily Rober, Kristee Brown, Kristena Y. Abdelmalak, Craig J. Galban, Timothy L. Frankel, Yaqing Zhang, Marina Pasca di Magliano, Stefanie Galban

**Affiliations:** 1 Graduate Program in Cancer Biology,; 2 Department of Radiology, and; 3 Center for Molecular Imaging, The University of Michigan Medical School, Ann Arbor, Michigan, USA.; 4 The Institute of Biorobotics, Scuola Superiore Sant’Anna, Pisa, Italy.; 5 Department of Surgery, The University of Michigan Medical School, Ann Arbor, Michigan, USA.; 6 PhD School in Translational Medicine, Scuola Superiore Sant’Anna, Pisa, Italy.; 7 Department of Biomedical Engineering,; 8 Rogel Cancer Center, and; 9 Department of Cell and Developmental Biology, The University of Michigan Medical School, Ann Arbor, Michigan, USA.

**Keywords:** Oncology, Cellular immune response, Lung cancer, Oncogenes

## Abstract

Lung cancer is the leading cause of cancer deaths in the United States. New targeted therapies against the once-deemed undruggable oncogenic KRAS are changing current therapeutic paradigms. However, resistance to targeted KRAS inhibitors almost inevitably occurs; resistance can be driven by tumor cell–intrinsic changes or by changes in the microenvironment. Here, we utilized a genetically engineered mouse model of KRAS^G12D^-driven lung cancer that allows for inducible and reversible expression of the oncogene: activation of oncogenic KRAS^G12D^ induces tumor growth; conversely, inactivation of KRAS^G12D^ causes tumor regression. We showed that in addition to regulating cancer cell growth and survival, oncogenic KRAS regulated the transcriptional status of cancer-associated fibroblasts and macrophages in this model. Utilizing ex vivo approaches, we showed that secreted factors from cancer cells induced the expression of multiple cytokines in lung fibroblasts, and in turn drove expression of immunosuppressive factors, such as arginase 1, in macrophages. In summary, fibroblasts emerged as a key source of immune regulatory signals, and a potential therapeutic target for improving the efficacy of KRAS inhibitors in lung cancer.

## Abstract 2

An oncogenic Kras-induced secretome drives immune suppression and supports tumor growth in lung adenocarcinoma by regulating fibroblasts in the tumor microenvironment.

## Introduction

In 2024, an estimated 234,580 new patients will be diagnosed with lung cancer in the United States, and 80% of those will have non–small cell lung cancer (NSCLC) (1). NSCLC is subclassified into 3 major groups: adenocarcinomas (40%), squamous cell carcinomas (30%), and large cell carcinomas (15%). KRAS is commonly mutated in adenocarcinomas. The most common point mutations are G12C (39%), followed by G12V (21%), G12D (17%), and G12A (10%) (2). Notably, while KRAS^G12C^ is the most prevalent mutation in adenocarcinoma among former or current smokers (42%), KRAS^G12D^ is most common in patients who have never smoked (56%) (2). Nonsmokers have poorer prognosis with worse outcomes to immunotherapies than smokers (3), possibly due to lower mutation burden — and thus, lower neoantigen prevalence and fewer changes in the tumor microenvironment.

While mutant KRAS was long deemed undruggable, several isoform-specific inhibitors have recently entered the clinic. The KRAS^G12C^ inhibitors sotorasib and adagrasib have gained FDA approval for lung cancer, while MRTX1133, a KRAS^G12D^ inhibitor, has entered phase I/II clinical testing for malignancies, including G12D-mutant lung cancer. While these are exciting developments, the prospect of resistance to targeted therapy is real; resistance already has been described for G12C inhibitors (4), with some instances involving sotorasib linked to the remodeling of the tumor microenvironment (5, 6). The effects of KRAS^G12D^ inhibition in lung adenocarcinoma, including changes in the tumor microenvironment and mechanisms of resistance, remain largely unknown.

Genetically engineered mouse models (GEMMs) expressing oncogenic KRAS upon Cre recombination, with or without mutation of the tumor suppressor p53, model the development of NSCLC resembling the human disease and are widely used for both basic discovery and preclinical studies (7). Studies using a similar model that allows both inducible and reversible expression of oncogenic KRAS^G12D^ have revealed that this oncogene is required not only for tumor initiation, but also for tumor maintenance, independently of the presence of additional mutations (such as inactivation of *Trp53* or other tumor suppressor genes) (8). However, the effect of oncogenic KRAS expression and subsequent inactivation in the tumor microenvironment in the lung remains unexplored. Given the likely need to devise combination targeting modalities to prevent or overcome resistance to targeted KRAS inhibitors, it is imperative to unravel the cellular crosstalk between KRAS-mutant epithelial cells and their surrounding microenvironment.

To study the extrinsic role of oncogenic KRAS in lung cancer maintenance and progression, we modified a GEMM that expresses mutant KRAS^G12D^ in an inducible and reversible manner in the lung epithelium. In this Lung-iKRAS (L-iKRAS) model, first described by the Varmus laboratory (8), expression of oncogenic KRAS in club cells, in combination with loss-of-function mutations in tumor suppressor genes, efficiently drives formation of lung adenocarcinoma in both male and female mice. Inactivation of oncogenic KRAS^G12D^ in tumors results in tumor regression, establishing a role for oncogenic KRAS^G12D^ in the maintenance of NSCLC.

We have re-derived the L-iKRAS model, incorporating current understanding of lung cancer etiology (9–11). While the initial model used a *Trp53*-null allele or *Ink4a*-null allele (8), we used an allele expressing mutant *Trp53^R172H^* — as mutant alleles are more common than null ones in human cancer — in an inducible manner (12), wherein mutant-p53 expression in the lung is activated by intranasal administration of adenovirus expressing Cre recombinase (ad-Cre) (8, 13). Here, we used the L-iKRAS model to drive NSCLC in mouse lungs; we then inactivated oncogenic KRAS and, as expected, observed tumor regression. Interestingly, characteristics of the microenvironment, such as fibroblast activation status, drastically changed upon KRAS inactivation. To gather an unbiased understanding of the effects of inactivating oncogenic KRAS, we performed single-cell RNA sequencing (scRNA-seq) on lungs of iKRAS mice with KRAS^G12D^ on (L-iKRAS^G12D^ ON) or lungs where KRAS^G12D^ had been inactivated (L-iKRAS^G12D^ OFF). In the presence of oncogenic KRAS, macrophages in the lung expressed immunosuppressive markers, consistent with the poor immunogenicity of KRAS^G12D^-mutant tumors. To understand how oncogenic KRAS regulates the composition and differentiation status of macrophages, we used ex vivo approaches. Mechanistically, we show that fibroblasts are a key signaling hub, mediating interactions between epithelial cells and macrophages, and thus, potentially mediating the immunosuppressive status of tumors.

## Results

### Inhibition of KRAS^G12D^ results in tumor regression in L-iKRAS mouse model of lung adenocarcinoma.

**Figure 1:**
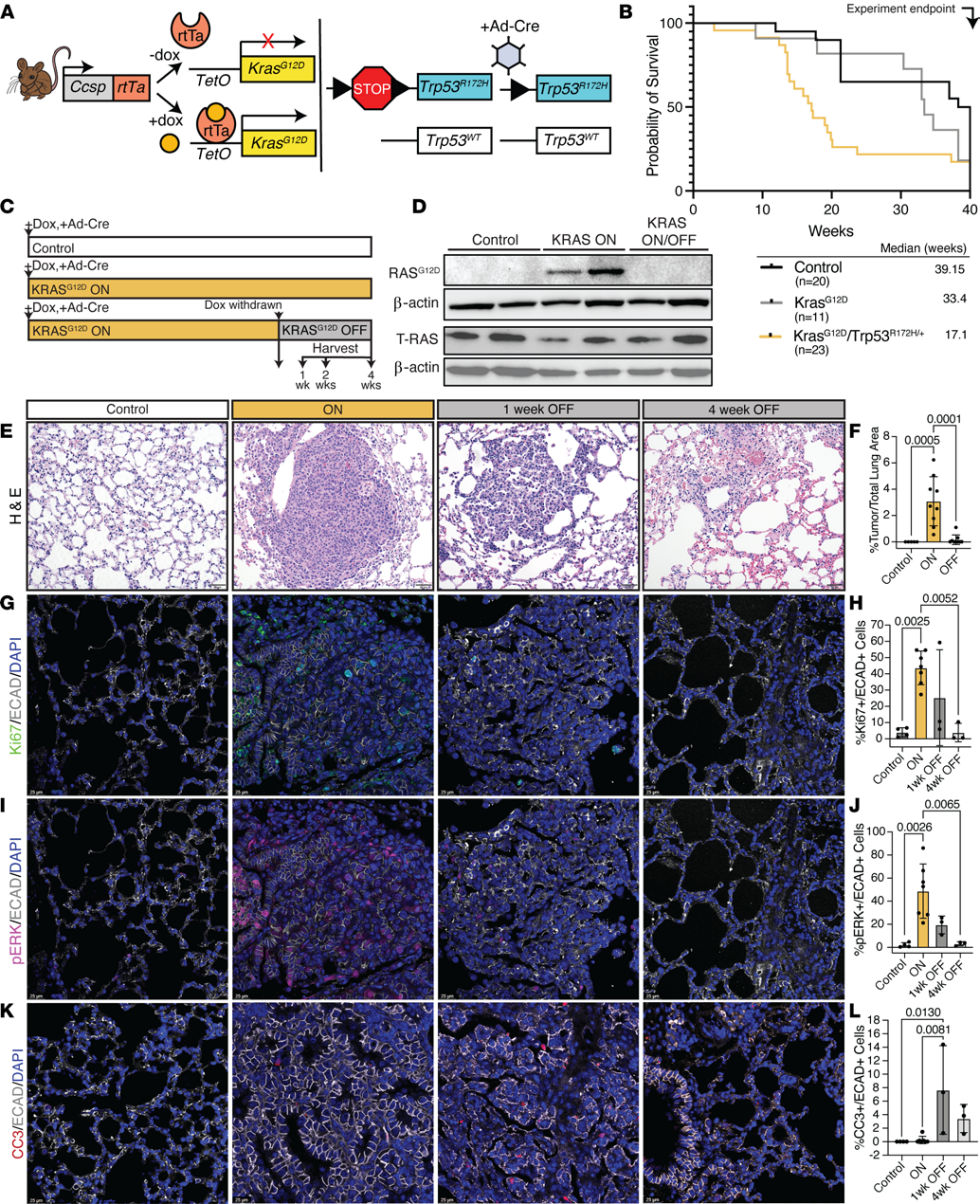
Inducible and reversible L-iKRAS^G12D^ mouse model of lung adenocarcinoma. (**A**) Schematic depicting L-iKRAS^G12D^ mouse model and its inducibility and reversibility of KRAS^G12D^ expression in club cells of the lung with dox and activation of mutant *Trp53* expression by ad-Cre. (**B**) Kaplan-Meier survival analysis comparing control mice (*n* = 20, *Ccsp-rtTa* or *TetO-Kras^G12D^* with or without *Trp53^–/+^* allele and with and without dox or ad-Cre), *Kras^G12D^* (*n* = 11, *Ccsp-rtTa*; *TetO-Kras^G12D^*; *Trp53^–/+^* on dox, but no ad-Cre or *Ccsp-rtTa*; *TetO-Kras^G12D^* on dox, with or without ad-Cre), and *Kras^G12D^*/*Trp53^R172H/+^* (*n* = 23, *Ccsp-rtTa*; *TetO-Kras^G12D^*; *Trp53^LSL-R172H/+^* on dox plus ad-Cre). Log-rank (Mantel-Cox) test with statistically significant *P* value of 0.0033. Median survival for all groups is indicated in inset. One-way ANOVA with Tukey’s post hoc test showed the median survival of the *Kras^G12D^*/*Trp53^R172H/+^* group was significantly lower than that of the control group (*P* value: 0.0014) and the *Kras^G12D^* group (*P* value: 0.0148). (**C**) Timeline for KRAS^G12D^ induction (ON) and KRAS^G12D^ inhibition (OFF) in triple-transgenic and control mice (single transgenics). (**D**) Western blot using anti-KRAS^G12D^ antibody or anti–total RAS (T-RAS) with corresponding β-actin blots as loading control of L-iKRAS lung tissue from all groups (ON: 20 weeks, OFF: 20 weeks ON and 4 weeks OFF). (**E**) Representative images of H&E. Scale bars: 50 mM. ON: 17–20 weeks, OFF 1 and 4 weeks. (**F**) Quantification of percentage tumor area over total lung area from whole slide scanned images in control, ON (17–25 weeks) and OFF (1, 2, and 4 weeks combined). (**G**) Representative images of Ki67/ECAD/DAPI. Scale bars: 25 mM. (**H**) Quantification of percentage Ki67^+^ cells among total ECAD^+^ cells. (**I**) Representative images of p-ERK/ECAD/DAPI. Scale bars: 25 mM. (**J**) Quantification of percentage p-ERK^+^ cells among total ECAD^+^ cells. (**K**) Representative images of CC3/ECAD/DAPI. Scale bars: 25 mM. (**L**) Quantification of percentage CC3^+^ cells among total ECAD^+^ cells. Data in **F**, **J**, **H**, and **L** are presented as mean ± SEM.

To generate a mouse model wherein oncogenic KRAS can be induced and reversed in the lung with accompanying mutant-p53 expression, we crossed the bitransgenic *Ccsp-rtTa*; *TetO-Kras^G12D^* mouse (8) with the *Trp53^LSL-R172H^* mouse (8, 14). In this study, we refer to this GEMM as L-iKRAS (*Ccsp-rtTa*; *TetO-Kras^G12D^*; *Trp53^LSL-R172H/+^*). Administration of doxycycline (dox) in drinking water induces expression of the reverse tetracycline transactivator (rtTa) in club cells of the lung, where it binds to the response element (*TetO-Kras^G12D^*) to initiate lung-specific expression of KRAS^G12D^. When dox is withdrawn from drinking water, rtTa is inactivated and expression of KRAS^G12D^ is prevented/reversed in club cells. Conditional, lung-specific expression of mutant p53^R172H^ in the lung can be achieved simultaneously by intranasal administration of ad-Cre at study start (Figure 1A). Single-transgenic mice given dox at the same concentration and for the same duration as experimental mice, or triple-transgenic mice that did not receive dox, served as controls for this study. Controls also included single- or triple-transgenic mice given ad-Cre. We compared overall survival of the 3 experimental cohorts to understand how KRAS^G12D^ expression alone, or KRAS^G12D^ with mutant p53 co-occurring mutation, impacts tumor growth in the lung (Figure 1B). The first experimental group (*Ccsp-rtTa*;*TetO-Kras^G12D^* with or without *Trp53^LSL-R172H/+^*) was given dox, but not ad-Cre, to induce expression of KRAS^G12D^ but not mutant p53. The control group, which included single-transgenic mice or *Trp53^LSL-R172H/+^* mice lacking a *Ccsp-rtTa* or *TetO-Kras^G12D^* cassette, was given dox and ad-Cre for a period of 40 weeks. The second experimental group, a triple-transgenic cohort (*Ccsp-rtTa*; *TetO-Kras^G12D^*; *Trp53^LSL-R172H/+^*), was given dox and ad-Cre at study start to induce KRAS^G12D^ and mutant p53 expression. Mice expressing only KRAS^G12D^ had a median survival of 33 weeks, whereas mice expressing both oncogenic KRAS and mutant p53 had a median survival of only 17 weeks, with survival decreasing after week 10. Differences in overall survival were statistically significant between all groups. The decreased survival of the mutant KRAS and mutant p53 groups may indicate that disease severity increases when p53 is co-mutated, as well as a transition from adenomas to adenocarcinomas (Figure 1B). To investigate the effect of KRAS^G12D^ inhibition on tumor growth, L-iKRAS ON mice and control littermates (Control) were given ad-Cre and dox water for 17–25 weeks. Fisher et al. showed that at 17–25 weeks, mice develop a measurable tumor burden, thus providing rationale for our analyses (8). A second group, L-iKRAS OFF mice, was given ad-Cre for 17 weeks before dox withdrawal for 1, 2, or 4 weeks (Figure 1C). We assessed lung sections from each group for KRAS^G12D^ expression by Western blotting using a RAS^G12D^-specific antibody. As expected, we detected RAS^G12D^ expression only in lung tissue from the ON group (Figure 1D). Next, we harvested lungs from all experimental cohorts to assess pulmonary lesions on hematoxylin and eosin–stained (H&E-stained) lung sections (Figure 1E). The percentage tumor area per lung area and the total number of lesions per lobe increased in the ON compared with the Control group and decreased statistically significantly when KRAS^G12D^ expression was reversed by dox removal for 1, 2, or 4 weeks (Figure 1F and Supplemental Figure 1A; supplemental material available online with this article; https://doi.org/10.1172/jci.insight.182228DS1, respectively). Higher magnification images of H&E staining are presented in Supplemental Figure 1B. Furthermore, depletion of oncogenic KRAS decreased cell proliferation in E-cadherin^+^ (ECAD^+^) epithelial cells, as assessed by Ki67 staining in all groups (Figure 1G, higher magnification in Supplemental Figure 1C; quantified in Figure 1H). In lung sections stained with an anti–p-ERK1/2 antibody, p-ERK1/2 expression increased in ECAD^+^ epithelial cells in the ON group and decreased in the OFF groups (Figure 1I, higher magnification in Supplemental Figure 1D; quantified in Figure 1J). Pulmonary lesions disappeared in the OFF groups, likely because of apoptotic cell death described by Fisher et al. (8) and assessed here by cleaved caspase 3 (CC3) staining in ECAD^+^ epithelial cells (Figure 1K, higher magnification in Supplemental Figure 1E; quantified in Figure 1L).

We also assessed inhibition by MRTX1133 (hereafter denoted MRTX), a clinically relevant KRAS^G12D^ inhibitor, as MRTX and other KRAS^G12D^ inhibitors are now in clinical trials. We orthotopically implanted KRAS^G12D^-mutant KPL-86 cells derived from the KP model into the lungs of mice, and then treated mice with MRTX for 2 days at 30 mg/kg twice daily by i.p. injection (see schematic in Supplemental Figure 1F). Lungs were harvested and stained with H&E, Ki67, p-ERK1/2, and CC3 (Supplemental Figure 1, G–I). Ki67 and p-ERK1/2 staining indicated that MRTX treatment resulted in a decrease in proliferation and MAPK signaling. Furthermore, apoptotic cell death increased in MRTX-treated lung sections compared with vehicle-treated sections. In summary, we demonstrate that induction of KRAS^G12D^ and co-occurring mutant p53 expression in the lung produces highly proliferative lung tumors that can be reduced by abrogating KRAS^G12D^ expression, genetically or chemically, resulting in fewer pulmonary lesions, likely due to apoptotic cell death of tumor cells.

### Oncogenic KRAS results in gene expression changes in epithelial cells in the tumor.

**Figure 2:**
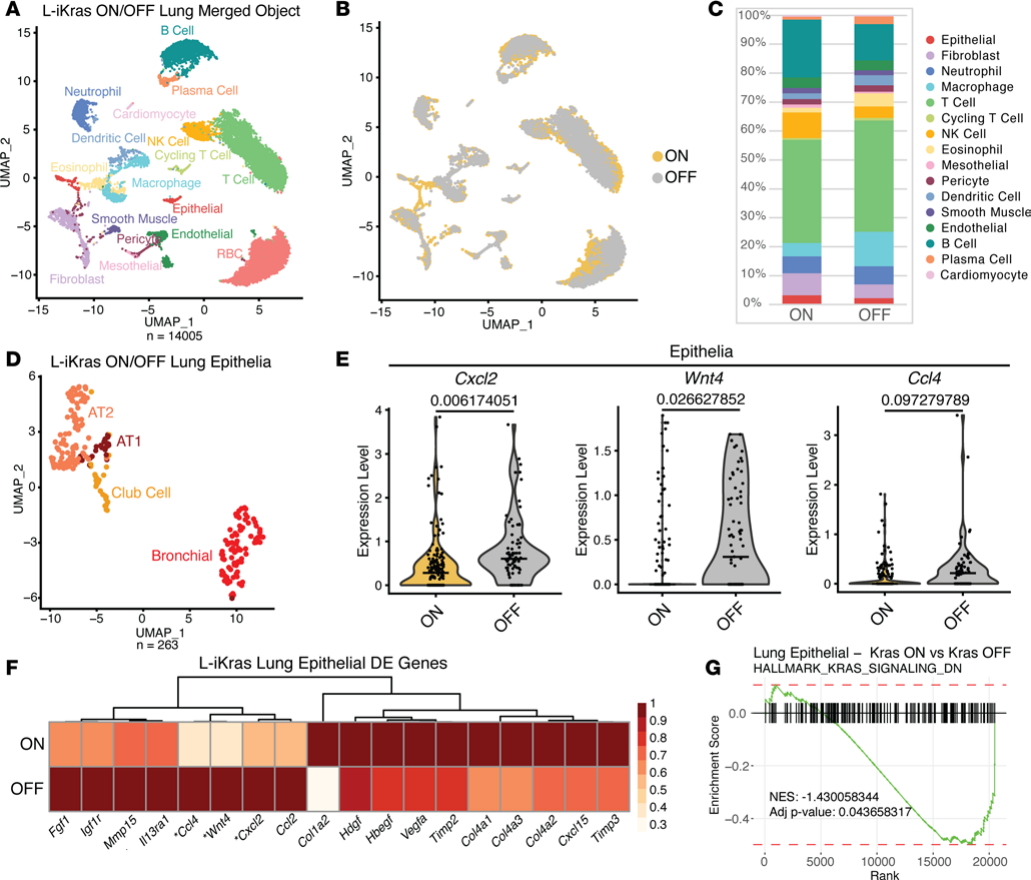
Oncogenic KRAS changes epithelial gene expression in the tumor microenvironment. (**A**) UMAP visualization of scRNA-seq data showing unsupervised clustering of cells from L-iKRAS lung samples (KRAS ON = 21 weeks ON dox; KRAS OFF = 20 weeks ON dox + 1 week OFF). Each color represents a distinct cell cluster. (**B**) UMAP visualization of scRNA-seq data showing overlap of KRAS ON and KRAS OFF groups. (**C**) Bar graph comparing cell cluster breakdown per sample (red blood cells were removed). (**D**) UMAP visualization of defined lung epithelial clusters from KRAS ON and KRAS OFF samples. (**E**) Violin plots of *Cxcl2*, *Wnt4*, and *Ccl4* comparing expression levels between total epithelial cells from KRAS ON and KRAS OFF samples. Adjusted *P* value given above each violin plot. Median expression is indicated with a horizontal line. (**F**) Heatmap showing averaged scRNA-seq expression data (relative to the highest expressor) for genes in epithelial cells curated from the differential gene expression list. Genes from **E** are marked with an asterisk. (**G**) GSEA plot of KRAS ON versus KRAS OFF lung epithelia showing the running enrichment score for the “HALLMARK_KRAS_SIGNALING_DN” gene set. Normalized enrichment score (NES) = –1.430058344. Adjusted *P* value = 0.043658317.

To better understand KRAS^G12D^-dependent changes occurring in the tumor and tumor microenvironment, we utilized scRNA-seq. We harvested the right inferior lobe of lungs from male and female mice in the ON (21 weeks) and OFF (21 weeks ON, 1 week OFF) groups. Samples were processed simultaneously to avoid batch effects. The processed scRNA-seq dataset had 14,005 cells: 9,090 from the ON group and 4,915 from the OFF group. Data were analyzed using the Seurat package in R (version 4.0.2) and visualized by uniform and manifold approximation and projection (UMAP) (Figure 2A). We observed no differences between male and female mice. As shown in Figure 2B, clusters from the ON and OFF groups overlaid sufficiently, indicating that similar cell types were present in both groups. Of note, we observed a higher percentage of fibroblasts in the ON compared with the OFF group and a higher percentage of macrophages in the OFF compared with the ON group (Figure 2C). To parse differences between groups in lung epithelia, we subclustered epithelial cells separately and visualized clusters by UMAP (Figure 2D). We assessed changes in gene expression in the epithelia; interestingly, genes upregulated in the OFF group included chemokines *Cxcl2* and *Ccl4*; *Wnt4*, a noncanonical WNT signaling ligand; growth factor *Fgf1*; and the extracellular matrix remodeling factor *Timp3*. Genes upregulated in the ON group included *Vegfa* and *Cxcl15* (Figure 2, E and F, and Supplemental Figure 2A). We also assessed expression changes for genes that change during lung cancer progression, including *Cxcl1*, *Tgfa*, and *Tgfb1*. While *Cxcl1* expression was upregulated in the OFF group, *Tgfa* expression was higher in the ON group; *Tgfb1* expression showed no change (Supplemental Figure 2B). Using gene set enrichment analysis (GSEA), we confirmed that KRAS signaling was efficiently abrogated in the OFF group, as the “HALLMARK_KRAS_SIGNALING_DN” gene set, a list of genes that are downregulated upon KRAS expression, was significantly upregulated in the OFF group (Figure 2G). Conversely, the “HALLMARK_KRAS_SIGNALING_UP” gene set, a list of genes that are upregulated upon KRAS expression, trended up in the ON group (Supplemental Figure 2C).

### Oncogenic KRAS results in gene expression changes in fibroblasts in the tumor microenvironment.

**Figure 3:**
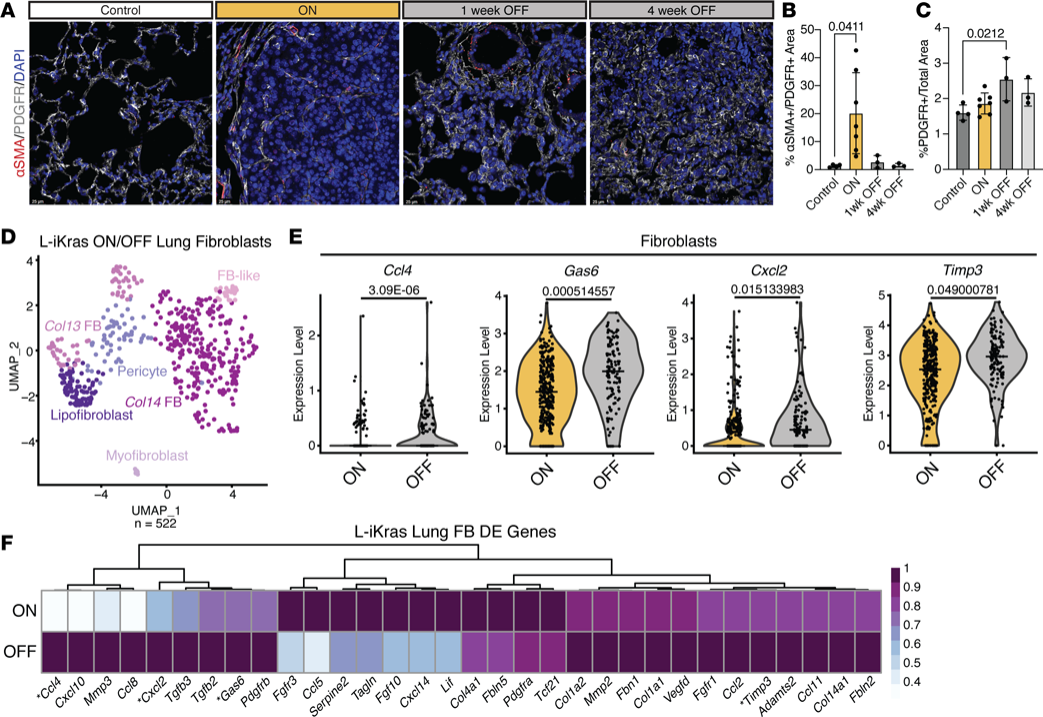
Oncogenic KRAS changes fibroblast gene expression in the tumor microenvironment. (**A**) Representative images of αSMA/PDGFR/DAPI. Scale bars: 25 mM. (**B**) Quantification of percentage αSMA^+^ area of total PDGFR^+^ area. (**C**) Percentage PDGFR^+^ area of total area. (**D**) UMAP visualization of scRNA-seq data showing unsupervised clustering of subsetted fibroblasts from KRAS ON and KRAS OFF samples. (**E**) Violin plots of *Ccl4*, *Gas6*, *Cxcl2*, and *Timp3* comparing expression levels between total epithelial cells from KRAS ON and KRAS OFF samples. Adjusted *P* value given above each violin plot. Median expression is indicated with a horizontal line. (**F**) Heatmap showing averaged scRNA-seq expression data (relative to the highest expressor) for genes in fibroblasts (pericytes removed) curated from the differential gene expression list. Genes from **E** are marked with an asterisk.

Fibroblasts play a key role in the development and progression of various cancers; specifically, cancer-associated fibroblasts (CAFs) interact with tumor and immune cells to mediate progression (for review see ref. 15). We performed immunofluorescent staining on lung sections from each experimental group from the L-iKRAS model to identify PDGFR^+^ fibroblasts in lung tumors. As depicted in Figure 3A and Supplemental Figure 3A, we identified PDGFR^+^ fibroblasts in all groups; more importantly, we showed that the activation status of these fibroblasts, as assessed by αSMA staining, increased in the ON group compared with control and decreased when KRAS^G12D^ was turned OFF, although total fibroblast numbers (PDGFR^+^ cells) did not change (Figure 3, B and C). To obtain mechanistic insight into fibroblast reprogramming, we subclustered the fibroblast population from the scRNA-seq data set and visualized it by UMAP (Figure 3D). We subclustered fibroblasts by comparing transcription of marker genes according to current literature (16, 17) (Supplemental Figure 3, B and C). Next, we looked at transcriptional differences between the ON and OFF groups through differential expression. Differentially expressed genes included cytokines and growth factors *Ccl4,* *Cxcl2*, *Tgfb*, and *Cxcl14*; extracellular matrix remodeling components *Timp3*, *Col4a1*, and *Fbln5*; and growth arrest–specific 6 (*Gas6*), which is upregulated during growth arrest (16, 17) (Figure 3, E and F, and Supplemental Figure 3D). Given the driver mutation in our mouse model, we also looked at genes that are upregulated in fibroblasts in KRAS^G12D^-driven pancreatic cancer (18). Interestingly, *Cxcl1* and *Il33* showed no change and *Il6* was upregulated in the OFF group (Supplemental Figure 3E). In contrast, in pancreatic cancer, oncogenic KRAS expression in epithelial cells drives an increase in fibroblast expression of both *Il33* and *Il6* (18, 19). Thus, the organ of origin determines the transcriptional program even in the presence of the same oncogene.

### Oncogenic KRAS regulates myeloid cells in the microenvironment of lung adenocarcinoma.

**Figure 4:**
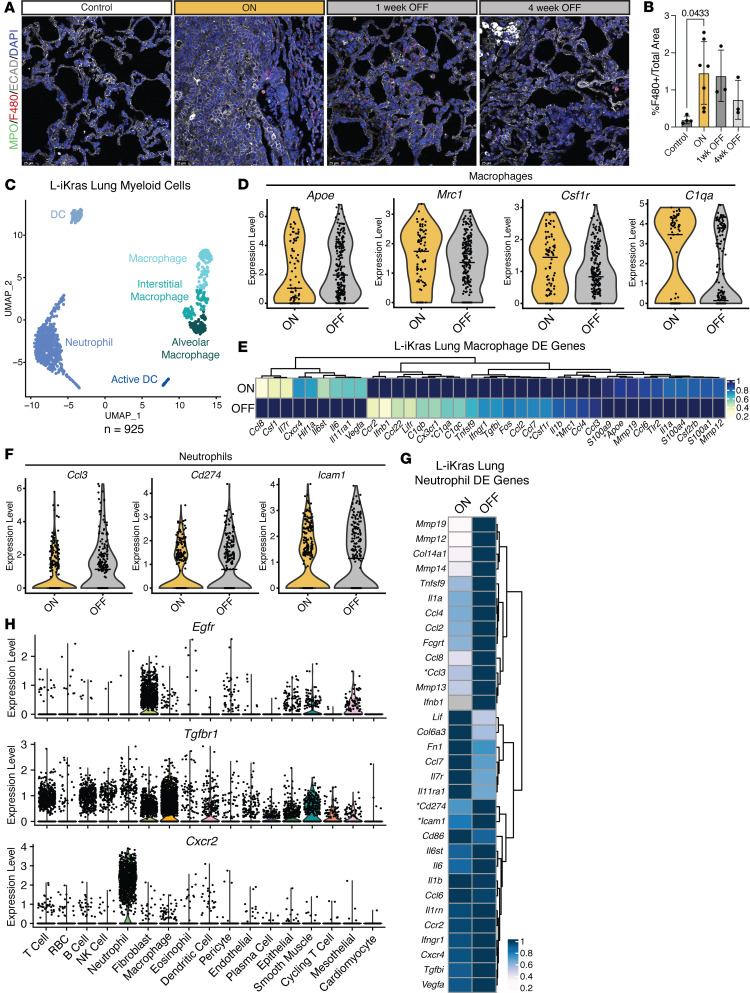
Oncogenic KRAS regulates myeloid compartment of the lung adenocarcinoma microenvironment. (**A**) Representative images of MPO/F4/80/ECAD/DAPI. Scale bars: 25 mM. (**B**) Quantification of percentage F4/80^+^ area of total area. (**C**) UMAP visualization of scRNA-seq data showing unsupervised clustering of subsetted myeloid cells from KRAS ON and KRAS OFF samples. (**D**) Violin plots of *Apoe*, *Mrc1*, *Csf1r,* and *C1qa* comparing expression levels between macrophages from KRAS ON and KRAS OFF samples. Median expression is indicated with a horizontal line. (**E**) Heatmap showing averaged scRNA-seq expression data (relative to the highest expressor) for genes in macrophages (combined macrophages, interstitial macrophages, and alveolar macrophages) curated from the differential gene expression list. Genes from **D** are marked with an asterisk. (**F**) Violin plots of *Ccl3*, *Cd274*, and *Icam1* comparing expression levels between neutrophils from KRAS ON and KRAS OFF samples. Median expression is indicated with a horizontal line. (**G**) Heatmap showing averaged scRNA-seq expression data (relative to the highest expressor) for genes in neutrophils from differential gene expression list. Genes from **F** are marked with an asterisk. (**H**) Violin plots showing expression of *Egfr*, *Tgfbr1*, and *Cxcr2* across all identified cell populations in KRAS ON and KRAS OFF samples combined.

To understand whether immune cells, specifically myeloid cells, in the tumor microenvironment of lung cancer are affected by oncogenic KRAS, we performed immunofluorescent staining on lung sections from each experimental group of the L-iKRAS mouse using macrophage marker F4/80 and neutrophil marker myeloperoxidase (MPO) (Figure 4A and Supplemental Figure 4A). Quantification of F4/80 and MPO showed a trend toward macrophages increasing in the ON group and decreasing in the OFF groups, albeit the finding was not statistically significant (Figure 4B and Supplemental Figure 4, B and C). These immune changes were distinct from what we observed in the orthotopic lung cancer model, where KP-derived cells (KPL-86) were implanted into the lung and mice treated with MRTX (Supplemental Figure 4D). In this case, macrophages appeared to decrease upon KRAS inactivation, while neutrophils increased.

Next, we queried scRNA-seq data to interrogate changes in myeloid cell composition by expression. We visualized changes by UMAP (Figure 4C), and found that *C1qa*, *C1qb*, and *C1qc*, immunosuppressive macrophage markers in pancreatic cancer (20), were upregulated in the ON compared with the OFF group (Figure 4D and Supplemental Figure 4E). Interestingly, *Apoe*, another marker of immunosuppression, was upregulated in the OFF group (Figure 4D). However, *Mrc1*, the gene encoding CD206, and *Csf1r* were both upregulated in the ON group, suggesting that overall, macrophages exhibit an immunosuppressive phenotype in this group (Figure 4D). Macrophages showed transcriptional changes, indicating increased immunosuppression upon oncogenic KRAS expression based on differential expression analysis (Figure 4E). Next, we interrogated differential expression changes in neutrophils and found that neutrophils in the OFF group increased both in cytokines such as *Ccl3* and polarization markers such as *Cd274* and *Icam1* (Figure 4, F–H). Interestingly, *Ccl3* and *Icam1* are markers of antitumoral neutrophils, whereas *Cd274* is a marker of activated neutrophils, indicating that turning oncogenic KRAS ON reprograms neutrophils to be less antitumoral (Figure 4G). This may provide insight into the efficacy of combining immune checkpoint inhibitor (ICI) therapies with KRAS inhibitors.

### Epithelial cell–derived secreted factors affect fibroblasts in a KRAS^G12D^-dependent manner.

**Figure 5:**
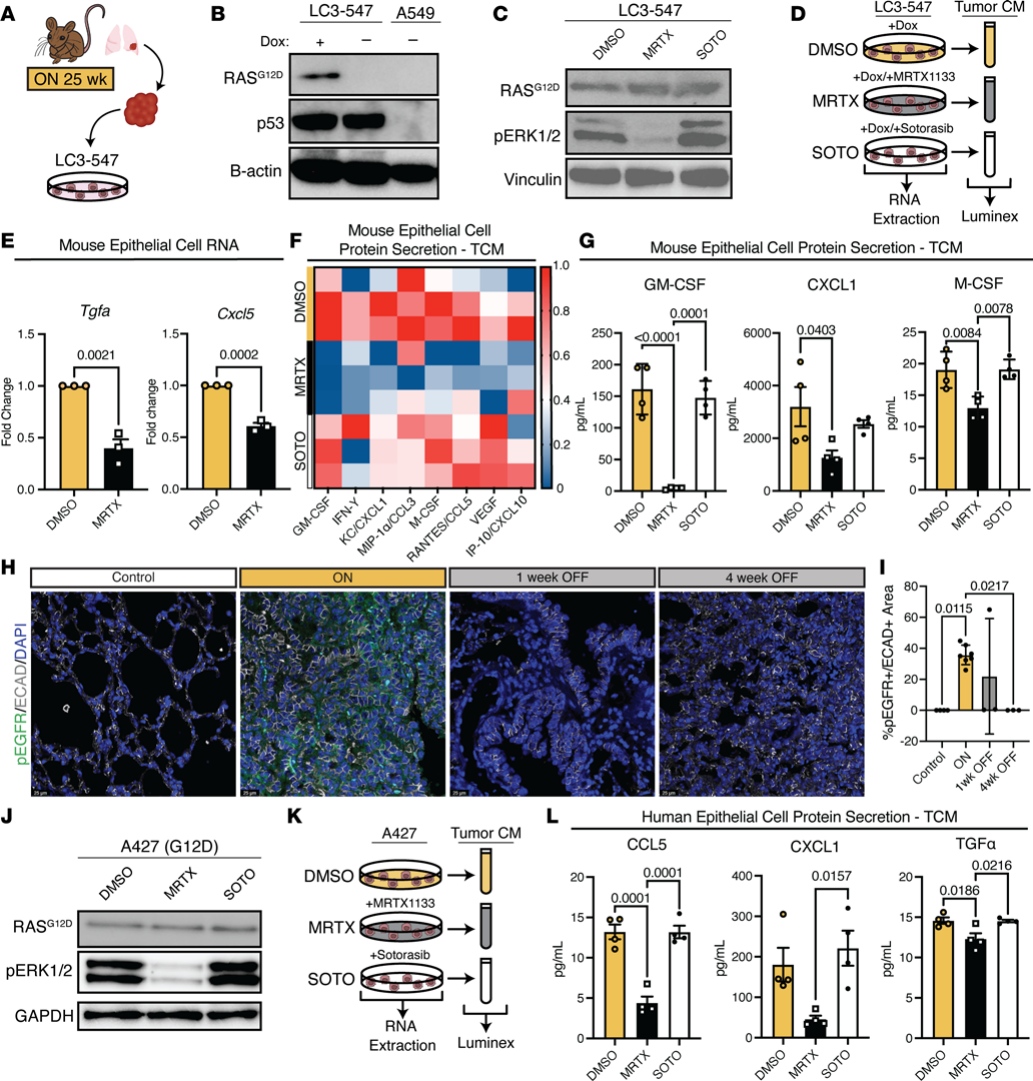
Identification of KRAS^G12D^-dependent immunosuppressive secretome. (**A**) Schematic depicting generation of LC3-547, an L-iKRAS cancer cell line from a murine lung tumor. (**B**) Representative Western blot depicting RAS^G12D^ and p53 protein expression in LC3-547 cells, which were cultured in dox-containing media for 24 hours prior to withdrawal of dox, and in A549 cells as controls. (**C**) Representative Western blot of RAS^G12D^ expression, p-ERK1/2, and vinculin in LC3-547 cells treated with 1 μM MRTX or 1 μM sotorasib (Soto) for 6 hours. (**D**) Depiction of the experimental outline for the collection of RNA and tumor-conditioned media (TCM) from LC3-547 cells treated with DMSO, MRTX, or Soto for subsequent analyses. All experiments were repeated at least 3 times, each time with 3 technical replicas. (**E**) qRT-PCR for *Tgfa* and *Cxcl5* of LC3-547 cells treated with DMSO or 500 nM MRTX for 48 hours. Data are represented as mean ± SEM, and statistical significance was determined with a 2-tailed Student’s *t* test for unpaired samples. (**F**) Heatmap with *z* score of Luminex data of TCM from LC3-547 cells treated with 500 nM MRTX, 500 nM Soto, or equimolar concentration DMSO for 48 hours depicting cytokine and growth factor secretion. (**G**) Quantification (pg/mL) of indicated cytokines in TCM from treated LC3-547 cells. Data are represented as mean ± SEM. Statistical significance was determined using 1-way ANOVA with Tukey’s post hoc test. (**H**) Representative images of p-EGFR/ECAD/DAPI. Scale bars: 25 mM. (**I**) Quantification of percentage p-EGFR^+^ area of total ECAD^+^ area. (**J**) Representative Western blot of KRAS^G12D^ expression and p-ERK1/2 in human KRAS^G12D^ lung adenocarcinoma cell line, A427, upon 3 hours treatment with 1 μM MRTX or 1 μM Soto or equimolar DMSO. (**K**) Experimental design: RNA and TCM were harvested from human KRAS^G12D^ cancer cells cultured with DMSO, 100 nM MRTX, or 500 nM Soto. All experiments were repeated at least 3 times, each time with 3 technical replicas. (**L**) Quantification of CXCL1, CCL5, and TGF-α cytokines in A427 TCM from cells treated with DMSO, MRTX, or Soto. Data are represented as mean ± SEM and differences were evaluated by 1-way ANOVA with post hoc Tukey’s HSD test.

First, we generated primary lung cancer cell lines from pulmonary lesions of L-iKRAS mice that received dox in drinking water for 25 weeks and ad-Cre at study start (Figure 5A). Cell lines were tested and selected for subsequent experiments if RAS^G12D^ and p53 were expressed compared with negative control (A549, human KRAS^G12S^), as seen in Figure 5B. We selected the L-iKRAS cell line LC3-547 because adding dox to media resulted in sustained expression of KRAS^G12D^ that could be reversed by dox removal or chemically inhibited by MRTX (Figure 5, B and C). We used the human KRAS^G12S^-expressing lung cancer cell line A549 as a negative control to demonstrate RAS^G12D^ antibody specificity (Figure 5B). We also tested sotorasib, a widely used FDA-approved KRAS^G12C^ inhibitor (hereafter denoted as “Soto”), in murine LC3-547 cells. We included Soto in subsequent experiments as an unspecific compound control; as expected, Soto did not affect downstream signaling such as phosphorylation of ERK1/2 in KRAS^G12D^ cells when compared with MRTX (Figure 5C). We next set up a cell culture system (Figure 5D), wherein cell lysates and conditioned media (CM) were collected from the different treatment groups, MRTX or Soto, to assess transcriptional activation and secretion of cytokines and growth factors modulated in our scRNA-seq data set upon KRAS^G12D^ activation (see Figure 2). To evaluate transcriptional changes in cytokines and growth factors induced by MRTX, we treated L-iKRAS cells (LC3-547) with MRTX, collected cell lysates, and analyzed RNA for expression of *Tgfa* and *Cxcl5* (Figure 5E). As we observed in the scRNA-seq data, both *Tgfa* and *Cxcl5* expression decreased upon chemical inhibition of KRAS. Furthermore, we observed these MRTX-induced transcriptional changes in LC3-547 cells when dox was removed (OFF) (Supplemental Figure 5A). We confirmed these changes in 2 additional lung cancer cell lines, derived either from the L-iKRAS model (LC3-545), or from the KP GEMM (KPL-86), depicted in Supplemental Figure 5, B and C, respectively. To compare KRAS^G12D^ protein expression in L-iKRAS–derived cells transcribed from the *TRE-Kras^G12D^* transgene to protein levels achieved from transcription of the endogenous locus in the widely used KP model (21), we tested RAS^G12D^ expression and downstream phosphorylation of ERK in KPL-86 cells side by side with LC3-547 cells (Supplemental Figure 5D). RAS^G12D^ expression in LC3-547 cells was similar or slightly less than in KPL-86 cells. Inhibition of KRAS^G12D^ with MRTX was equivalent in both lines, as tested by surrogate phosphorylation of ERK1/2 (Supplemental Figure 5D).

Next, we treated LC3-547 cells grown in media containing dox with DMSO, MRTX, or Soto to obtain tumor-conditioned media (TCM) from all treatment groups (ON, MRTX, Soto) for multiplex ELISA (Luminex) analysis. The heatmap in Figure 5F depicts a clear difference in cytokine/growth factor secretion between DMSO- and MRTX-treated cells; as expected, Soto, the unspecific compound control, had no effect and resembled the cytokine/growth factor secretion of the DMSO-treated cells. Selected growth factors and cytokines like GM-CSF, CXCL1, and M-CSF showed statistically significant decreased secretion when treated with MRTX, but not with Soto (Figure 5G). Since we showed KRAS^G12D^-dependent expression of *Tgfa* in L-iKRAS cells, we assessed downstream signaling activation through phosphorylation of EGFR, an important growth factor receptor in lung cancer, in lung sections from L-iKRAS mice. As seen in Figure 5H (higher magnification in Supplemental Figure 5E; quantification in Figure 5I), p-EGFR increased in the ON group and decreased in the 4 weeks OFF group. We confirmed these findings in lung sections from MRTX-treated orthotopically implanted mouse tumors (Supplemental Figure 5F).

To recapitulate these findings in human lung cancer cells, we treated the KRAS^G12D^-mutant lung adenocarcinoma cell line A427 with MRTX or Soto prior to collecting protein, RNA, and TCM (Figure 5, J and K). Figure 5J shows that A427 cells expressed KRAS^G12D^ and exhibited high levels of ERK1/2 phosphorylation, which markedly decreased when cells were treated with MRTX, but not Soto. The downstream effects of KRAS^G12D^ inhibition were sustained for more than 24 hours, as assessed by phosphorylation of ERK1/2 (Supplemental Figure 5G). Furthermore, we showed using quantitative real-time PCR (qRT-PCR) that expression of *TGFA*, *TGFB*, *CXCL1*, and *CXCL2* was reduced in MRTX-treated cells (Supplemental Figure 5H). TCM secretome analysis using Luminex assays revealed that secretion of CCL5, CXCL1, and TGF-α was KRAS^G12D^ dependent, as it was inhibited in MRTX-treated, but not Soto-treated cells (Figure 5L). Together, these data reveal an epithelial KRAS^G12D^-dependent secretome containing tumor-promoting and immunosuppressive components that may provide future therapeutic targets.

### KRAS^G12D^-dependent regulation of macrophages by CAFs.

**Figure 6:**
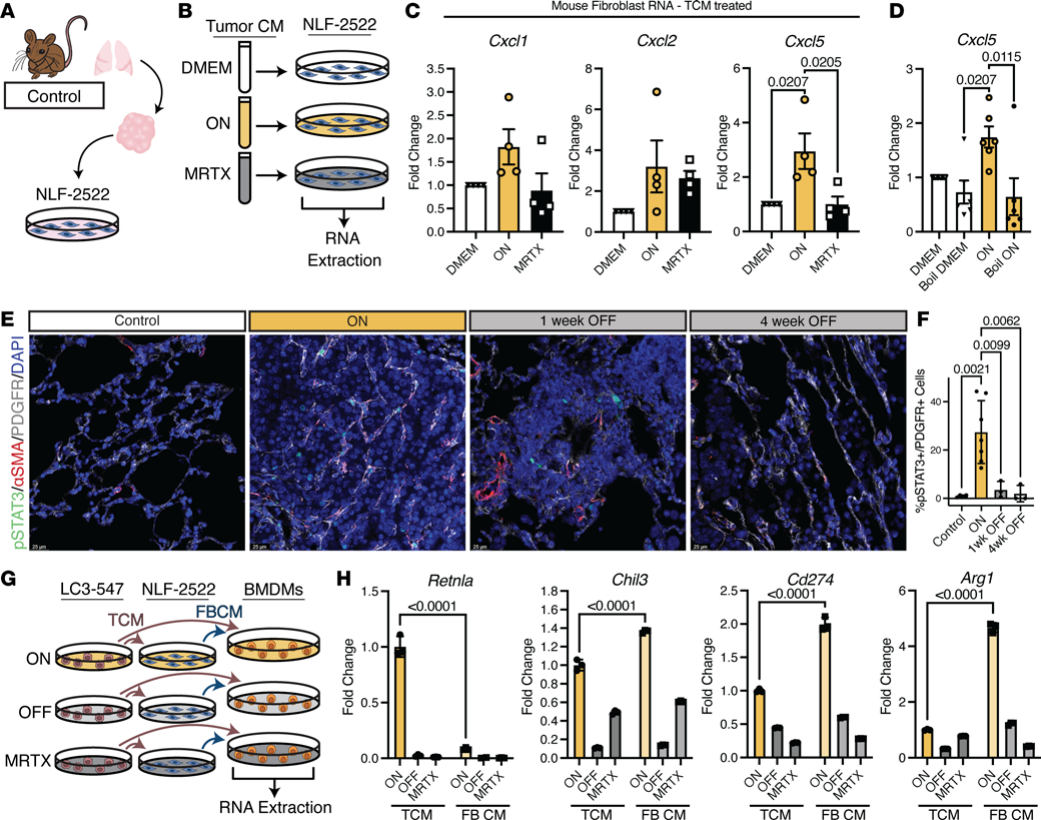
CAFs provide KRAS^G12D^-dependent immunosuppressive paracrine signals. (**A**) Schematic depicting derivation of normal lung fibroblasts, NLF-2522, from syngeneic L-iKRAS control mouse. (**B**) Experimental outline: lung fibroblasts were treated with TCM from ON, MRTX-treated LC3-547 cells, or DMEM alone as no-cell control. All experiments were repeated at least 3 times, each time with 3 technical replicas. (**C**) RNA was isolated from NLF-2522 fibroblasts incubated with TCM from ON or MRTX groups and expression of indicated genes was compared to DMEM-treated cells by qRT-PCR. Data are represented as mean ± SEM and statistical analysis was performed by 1-way ANOVA with post hoc Tukey’s HSD test. (**D**) TCM or DMEM was heated at 95°C–100°C for 10 minutes and subsequently added to NLF-2522 fibroblasts for 24 hours. Expression of *Cxcl5* was assessed by qRT-PCR and compared to cells incubated with non-boiled TCM or DMEM. (**E**) Representative images of p-STAT3/αSMA/PDGFR/DAPI. Scale bars: 25 mM. (**F**) Quantification of p-STAT3^+^ cells as percentage of total PDGFR^+^ cells. (**G**) Experimental design of BMDMs cultured in conditioned medium (CM) from ON, OFF, MRTX-, and DMEM-treated fibroblasts. All experiments were repeated at least 3 times, each time with 3 technical replicas. (**H**) qRT-PCR for expression of M2 markers *Retnla*, *Chil3*, *Cd274*, and *Arg1* in BMDMs treated with media from ON, OFF, and MRTX-treated L-iKRAS cells (TCM) or media from fibroblasts treated with ON, OFF, and MRTX-treated L-iKRAS cells (FB CM). Data are represented as mean ± SEM and statistical analysis was performed by 1-way ANOVA with post hoc Tukey’s HSD test.

CAFs support tumor growth through paracrine signaling. Here, we established an in vitro system to recapitulate paracrine signals identified in scRNA-seq analyses (see Figure 3). For this purpose, we established lung fibroblasts, hereafter termed NLF-2522, from our syngeneic L-iKRAS control mouse (Figure 6A). To investigate whether CAFs can be generated in TCM, we incubated NLF-2522 cells in TCM from LC3-547 cells from ON or MRTX-treated groups for 24 hours prior to isolating RNA for qRT-PCR analyses (Figure 6B). We identified statistically significant changes for the expression of *Cxcl5*; a similar trend, albeit not statistically significant, was identified for *Cxcl1* and *Cxcl2*, indicating that fibroblast phenotype and paracrine signals can change upon incubation with TCM (Figure 6C). Next, we asked whether dox removal similarly affects fibroblast gene expression. We cultured NLF-2522 fibroblasts in TCM from ON, OFF, and MRTX-treated LC3-547 cells for 24 hours prior to qRT-PCR analysis of *Cxcl1*, *Cxcl2*, and *Cxcl5*. Similar to MRTX, dox removal resulted in a reduction in cytokine expression (Supplemental Figure 6A). Furthermore, upon boiling TCM from the ON group, the effects on *Cxcl5* expression were lost, indicating that TCM factors are destroyed by heat exposure (Figure 6D). To recapitulate KRAS^G12D^-dependent paracrine signaling mechanisms stemming from human fibroblasts, we incubated CCL210 human adult lung fibroblasts in TCM from A427 cells treated with MRTX or equimolar concentrations of DMSO (Supplemental Figure 6B). We collected CM (FB CM) for secretome analysis of candidate cytokines. Cells were pelleted to obtain RNA for gene expression studies. Surprisingly, gene expression of *CXCL1*, *CXCL2*, and *CXCL5* did not change in human CAFs incubated with TCM from untreated or MRTX-treated A427 cells (Supplemental Figure 6C), but we did find that secretion of TGF-α, CXCL1, and CCL5 cytokines assessed by Luminex analysis was KRAS^G12D^ dependent (Supplemental Figure 6D). In fact, we observed a statistically significant reduction in TGF-α, CXCL1, and CCL5 secretion upon KRAS^G12D^ inhibition with MRTX (Supplemental Figure 6D); however, when comparing the amount of secreted cytokines in FB CM to TCM, we did not observe a statistically significant increase, indicating that fibroblasts likely do not produce these cytokines, a finding corroborated by qRT-PCR (Supplemental Figure 6C). To determine whether p-STAT3, an indicator of JAK/STAT3 signaling, is activated in lung fibroblasts, we stained lung sections from each experimental group from the L-iKRAS model for p-STAT3, αSMA, and PDGFR. As shown in Figure 6E (high magnification in Supplemental Figure 6E) and quantified in Figure 6F, p-STAT3 was activated in fibroblasts in the ON group, but reduced in the OFF groups, indicating dependence on extrinsic oncogenic KRAS. Similarly, we analyzed how the KRAS^G12D^ inhibitor MRTX affected fibroblast signaling by staining for p-STAT3 of lung sections obtained from mice with orthotopic KPL-86 lung tumors treated with vehicle or MRTX. As observed in the L-iKRAS model, upon inhibition of oncogenic KRAS, phosphorylation of STAT decreased (Supplemental Figure 6F).

To understand the consequence of KRAS^G12D^-dependent paracrine signaling from CAFs, we established another in vitro system utilizing bone marrow–derived macrophages (BMDMs). CXCL1 and CXCL5 regulate myeloid recruitment, activation, and polarization; to assess whether KRAS^G12D^-mediated TCM or fibroblast cytokine secretion plays a role in the polarization of macrophages, we isolated and differentiated BMDMs from control mice and cultured them in TCM or FB CM from ON, OFF, and MRTX-treated LC3-547 lung cancer cells or fibroblasts for 6 days (Figure 6G). Gene expression analysis performed on conditioned macrophages revealed statistically significant differences between expression of established tumor-associated macrophage (TAM) markers *Arg1*, *Rentla*, *Chil3*, and *Cd274* (22, 23) when BMDMs were cultured in ON TCM or FB CM. Interestingly, expression of *Chil3*, *Cd274*, and *Arg1* appeared to require additional factors stemming from CAFs; while their expression was induced by culture in FB CM, *Retnla* expression was reduced. Moreover, expression of these immunosuppressive TAM M2-like markers was dramatically reduced when BMDMs were cultured in OFF and MRTX in both TCM and FB CM (Figure 6H), indicating that CAFs may not be required for mediating KRAS inhibition.

To determine the relevance of CXCL signaling on macrophage polarization to TAMs, we incubated BMDMs with ON FB CM supplemented with an inhibitor (SB225002) against the cognate receptor for CXCL1, CXCL2, and CXCL5 (CXCR2) (Supplemental Figure 6G). Treatment with CXCR2 inhibitor SB225002 significantly prevented TAM marker expression, but did not abrogate expression entirely – as seen with MRTX – indicating that CXCL1, CXCL2, and CXCL5 are not the sole factors regulating polarization of macrophages to TAMs (Supplemental Figure 6G). In summary, our data suggest a mechanism by which CAFs may further mediate immunosuppression through CXCL1, CXCL2, and CXCL5 signaling in a KRAS^G12D^-dependent manner, as signals stemming from epithelial cells expressing oncogenic KRAS are required to activate fibroblasts.

### KRAS^G12D^-dependent crosstalk between epithelial cells, fibroblasts, and myeloid cells is recapitulated in human lung adenocarcinoma.

**Figure 7:**
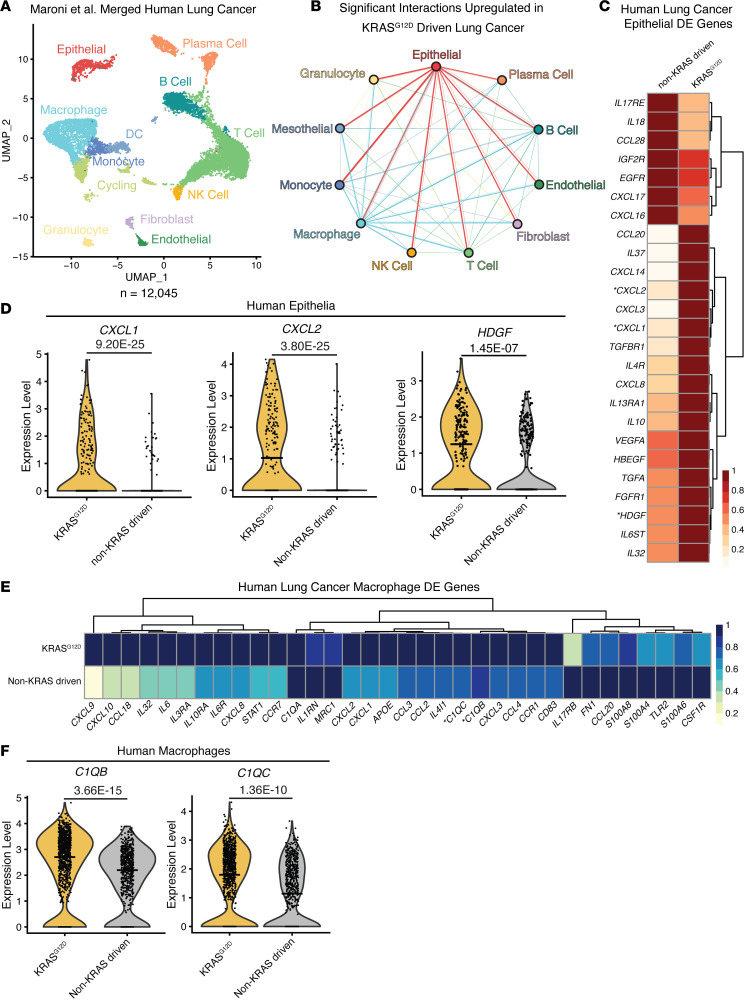
scRNA-seq analysis of human lung adenocarcinoma recapitulates gene expression of distinct immunosuppressive markers from L-iKRAS epithelial and fibroblast secretomes. (**A**) UMAP visualization of scRNA-seq data showing unsupervised clustering of cells from lung cancer samples. KRAS status: KRAS^G12D^ or wild-type KRAS (24). Each color represents a distinct cell cluster. (**B**) Interactome showing potential ligand/receptor pair interactions that are significantly (adjusted *P* value < 0.05) upregulated in KRAS^G12D^-driven lung cancer samples. (**C**) Heatmap showing averaged scRNA-seq expression data (relative to the highest expressor) for genes in epithelial cells curated from the differential gene expression list. Genes from **D** are marked with an asterisk. (**D**) Violin plots of *CXCL1*, *CXCL2*, and *HDGF* comparing expression levels between total epithelial cells from KRAS^G12D^ and KRAS–wild-type samples. Adjusted *P* value given above each violin plot. Median expression is indicated with a horizontal line. (**E**) Heatmap showing averaged scRNA-seq expression data (relative to the highest expressor) for genes in human macrophages (24) curated from the differential gene expression list. Genes from **F** are marked with an asterisk. (**F**) Violin plots of *C1QB* and *C1QC* comparing expression levels between total epithelial cells from KRAS^G12D^ versus KRAS–wild-type samples. Adjusted *P* value given above each violin plot. Median expression is indicated with a horizontal line.

To assess the clinical relevance of our findings in the KRAS inducible and reversible lung adenocarcinoma mouse model, we used a publicly available scRNA-seq dataset of KRAS^G12D^-driven and non–KRAS-driven lung cancer (24). We analyzed data similarly to our mouse scRNA-seq dataset (see Figure 2A), with the addition of batch correction for patients using Seurat’s IntegrateData pipeline (25). The dataset was visualized using UMAP (Figure 7A). To recapitulate crosstalk between cell types found in the tumor microenvironment of murine lung cancer, we filtered scRNA-seq data for expression of known signaling pairs (26). We identified significant predicted interactions upregulated in KRAS^G12D^-driven lung cancer (Figure 7B) that indicate epithelial cell and macrophage signaling changes the most. Differential expression analysis of epithelial cells showed a significant increase in expression of *CXCL1*, *CXCL2*, and *HDGF* in KRAS^G12D^-driven lung cancer samples compared with non–KRAS-driven lung cancer (Figure 7, C and D). Furthermore, we observed a significant increase in expression of *C1QB* and *C1QC* in macrophages in KRAS^G12D^-driven, but not non–KRAS-driven, lung cancer, recapitulating our findings in the L-iKRAS model (Figure 7, E and F). Although the number of fibroblasts in the human samples was limited, we corroborated some in vitro data, including increased expression of CXCL1 in the KRAS^G12D^-driven versus non–KRAS-driven lung cancer samples (Supplemental Figure 7A). These findings provide potential targets in the tumor microenvironment for future studies evaluating co-targeted approaches with KRAS inhibitors.

In summary, our findings lend mechanistic insight to the extrinsic signaling consequences of oncogenic KRAS in lung cancer. We have identified a KRAS^G12D^-dependent secretome that regulates and activates fibroblasts, which in turn recruit, activate, and polarize myeloid cells to form a tumor-promoting, immunosuppressive microenvironment. Furthermore, we present factors that are upregulated in response to KRAS inhibitors that potentially indicate relevant mechanisms of resistance.

## Discussion

Oncogenic mutations in the *KRAS* gene are a hallmark of many cancer types, including a subset of NSCLC (2). With the advent of isoform-specific mutant-KRAS inhibitors, understanding how oncogenic KRAS functions in the context of advanced disease is critical to prevent or overcome resistance to targeted treatment. Mutant KRAS is a driver in lung cancer (4); furthermore, it is required for tumor maintenance in a mouse model of the disease (8), indicating a cell-autonomous role of oncogenic KRAS in cancer cells. Less well understood is the extrinsic effect of modulating oncogenic KRAS on the tumor microenvironment. Here, using a model of inducible and reversible expression of oncogenic KRAS in the lung, we show that the fibroblast transcriptional program directly responds to signals from tumor epithelial cells. While we previously described similar findings in pancreatic cancer (18), this transcriptional reprogramming is organ specific; in pancreatic cancer, *Il33* and *Il6* are key factors upregulated in fibroblasts, but these are not upregulated in lung fibroblasts. However, other transcriptional programs activating cytokines in the *CCL* and *CXCL* families are common between the 2 malignancies, pointing to a yet poorly understood tissue-specific nature of fibroblasts.

Overall, in response to oncogenic KRAS expression in cancer cells, fibroblasts activate cytokines with cognate receptors expressed in immune cells, prevalently myeloid cells. Our experiments show that fibroblast factors activate *Arg1*, an immunosuppressive marker (27), in macrophages at a higher level than TCM alone. Together, our data point to fibroblasts amplifying epithelial signals and promoting the establishment of an immunosuppressive microenvironment.

Organ-specific differences may explain the clinical outcomes of lung and pancreatic cancers, including varied responses to immunotherapy. In pancreatic cancer, inactivation of oncogenic KRAS improves T cell infiltration; furthermore, combination therapy with KRAS inhibitors and immunotherapeutic agents improves responses and delays or prevents acquired resistance to KRAS inhibitors (28, 29). Future work is needed to identify the specific tumor factors that drive fibroblast reprogramming, and to test avenues to target fibroblasts to ameliorate immunosuppression in lung cancer.

Considerable heterogeneity in lung cancer has prevented efficacy of new targeted immune therapies, with only approximately 11% of all lung cancer patients responding to ICI therapy. Recent single-cell sequencing studies in human lung adenocarcinoma and murine KP models provide some insight into not only the heterogeneity of tumors, but also the composition of the tumor microenvironment (16, 24). Specifically, tumor-infiltrating immune cells, fibroblasts, and endothelial cells contribute to a distinct tumor microenvironment in lung adenocarcinoma and likely drive its progression. In accordance with some of our findings, Kim et al. have described molecular and cellular reprograming of metastatic lung adenocarcinoma and how stromal and immune interactions result in a protumoral and immunosuppressive microenvironment, which likely drives progression to metastatic disease (16). Furthermore, Kim and colleagues showed that lung-resident myeloid cells are gradually replaced with monocyte-derived macrophages and dendritic cells during progression, along with increased T cell exhaustion (16). Here, we provide insight into the gene expression changes initiated upon KRAS activation that result in the activation of CAFs in the surrounding stroma. Growth factors such as TGF-α and TGF-β have long been described as important factors in lung tumor growth, but it was unknown whether they were KRAS dependent. Activation of fibroblasts in turn results in expression and secretion of factors like CXCL1, CXCL2, and CXCL5, which recruit and polarize macrophages, but not in a KRAS-dependent manner. Inhibition of the receptor for these cytokines using a CXCR2 inhibitor provides further insight into the importance of fibroblast-dependent immune polarization. Our study thus provides mechanistic insight into how KRAS^G12D^ drives tumorigenesis in the lung and identifies potential actionable factors that could be utilized to target the tumor microenvironment in lung cancer.

In summary, resistance to KRAS inhibitors is anticipated with their increased clinical use, and the mechanisms of resistance need to be understood to provide long-term benefits for lung cancer patients. Very few mechanistic studies have interrogated changes in the tumor microenvironment upon KRAS inactivation or inhibition. Here, we look at changes occurring in the tumor microenvironment upon both activation and inactivation of oncogenic KRAS, including the role of fibroblasts and immune cells in the remodeling process. The L-iKRAS mouse provides a murine model system for further interrogating these observed changes to predict mechanisms of resistance and identify co-targeted strategies for future use with KRAS inhibitors. Thus, our findings provide rationale to co-target changes in the tumor microenvironment to prevent non-genetic mechanisms of resistance to KRAS inhibitors observed in roughly one-third of sotorasib-treated NSCLC patients (30), which will guide precision medicine for lung cancer patients.

## Methods

### Sex as a biological variable.

We utilized both male and female mice in our study and report similar findings for both sexes.

### Genetically engineered mouse model: L-iKRAS.

To re-derive the L-iKRAS model (8), we crossed *Ccsp-rtTa* and *TetO-Kras^G12D^* mice. *Ccsp-rtTa* mice were a gift from William D. Hardie (Cincinnati Children’s Hospital Medical Center, Cincinnati, Ohio, USA). Subsequently, we crossed *Ccsp-rtTa; TetO-Kras^G12D^* mice with *Trp53^LSL-R172H/+^* mice developed and described by Tyler Jacks (31). In the resulting L-iKRAS model (*Ccsp-rtTa; TetO-Kras^G12D^; Trp53^LSL-R172H/+^*), we induced KRAS^G12D^ expression by adding dox (Sigma-Aldrich, D9891) to drinking water at a concentration of 0.5 mg/mL and mutant p53 expression by 1-time intranasal instillation of ad-Cre recombinase with 3 × 10^7^ PFU/50 μL/mouse at study start. We reversed KRAS^G12D^ expression in the lungs of L-iKRAS mice at indicated time points by removing dox from drinking water. We included several control groups in our study: single-transgenic mice (*Ccsp-rtTa*) or (*TetO-Kras^G12D^*), which received the same concentration of dox in drinking water for the same duration as experimental mice; and L-iKRAS mice (*Ccsp-rtTa; TetO-Kras^G12D^; Trp53^LSL-R172H/+^*), which did not receive dox in drinking water. We also included *Ccsp-rtTa, TetO-Kras^G12D^* mice, which received ad-Cre, and L-iKRAS mice (*Ccsp-rtTa; TetO-Kras^G12D^; Trp53^LSL-R172H/+^*), which received 50 μL of saline for sham intranasal instillation. Mice were housed in a pathogen-free environment and cared for by the Unit for Laboratory Animal Medicine (ULAM) and the Galban laboratory.

### Orthotopic lung cancer model and in vivo dosing with MRTX.

We transduced KPL-86 cells with luciferase-expressing lentivirus (pLVX EF1α IRES blast) (32). In brief, 200,000 KPL-86-luc cells were implanted intracostally into the left lung. Mice were monitored by bioluminescence imaging (BLI); when BLI reached 1 × 10^7^ p/s at approximately 14 days after implantation, we randomized mice into 2 treatment groups and initiated treatment with vehicle (10% [w/v] sulfobutylether-β-cyclodextrin) or 30 mg/kg MRTX1133 (Selleckchem, E1051) in vehicle twice a day for 2 days by i.p. injection. Twelve hours after the last treatment, mice were sacrificed and lungs flushed with 1× PBS and fixed, paraffin embedded, sectioned, and stained as described below (see *Histology, immunofluorescence, and quantification*).

### scRNA-seq.

We harvested the right inferior lobe of the lung for scRNA-seq analysis from mice ON for 21 weeks (*n* = 2, pooled for submission) or ON for 20 weeks and then OFF for 1 week (*n* = 2, pooled for submission). Samples were processed simultaneously to avoid batch effects. Lung tissues were mechanically minced and enzymatically digested with collagenase P (1 mg/mL in RPMI 1640) at 37°C for 30 minutes. Cell suspensions were filtered through 500 μm, 100 μm, and 40 μm mesh cell strainers. Dead cells were removed using the MACS Dead Cell Removal Kit (Miltenyi Biotec, 130-090-101). Single-cell complementary DNA libraries were prepared and sequenced at the University of Michigan Advanced Genomics Core using the 10× Genomics Platform. Samples were run using 50-cycle, paired-end reads on the HiSeq 4000 (Illumina) to a depth of 100,000 reads. Raw data were processed and aligned by the University of Michigan Advanced Genomics Core. Cell Ranger count version 6.0.0 (10× Genomics) was used with default settings, with an initial expected cell count of 10,000. R version 4.2.1, RStudio version 2023.06.0+421, and R package Seurat version 4.0.2 were used for scRNA-seq data analysis (33–35). We initially filtered data to include cells with at least 100 genes and genes found in more than 3 cells. Data were normalized using the NormalizeData function with a scale factor of 10,000 and the LogNormalize normalization method. We then filtered data manually to include only cells with 800–100,000 transcripts and less than 15% mitochondrial genes. Variable genes were identified using the FindVariableFeatures function. Data were scaled and centered using linear regression of transcript counts. PCA was run with the RunPCA function using the previously defined variable genes. Cell clusters were identified via the FindNeighbors and FindClusters functions, using dimensions corresponding to approximately 90% variance as defined by PCA. UMAP clustering algorithms were performed with RunUMAP. Clusters were characterized by defined gene expression profiles. The complete R script, including figure-specific visualization methods, is publicly available on GitHub (https://github.com/Galban-Lab).

To analyze human single-cell data, we applied the same methods used for mouse data, with the addition of Seurat’s IntegrateData pipeline to mitigate batch effects (25). We utilized the following samples from the NCBI Gene Expression Omnibus (GEO) series GSE136246 (24): 2 KRAS–wild-type patient tumors (3 samples: NSC010.t1, NSC010.t2, and NSC037) and 3 KRAS^G12D^-mutant patient tumors (6 samples: NSC016.t1, NSC016.t2, NSC016.t3, NSC020.t1, NSC020.t2, and NSC036).

Interactome analysis was performed as described by Velez-Delgado et al. (18). We defined ligand-receptor pairs based on a curated, literature-supported list described by Ramilowski et al. (36) and further curated by Steele et al. (37). The average expression values of ligands and receptors in each cell population for both groups (KRAS^G12D^ and non–KRAS-driven) were calculated individually. Ligands and receptors that expressed below the median average expression or were not expressed in both experimental groups were excluded from analysis. Differences in ligands and receptors between groups were determined using Wilcoxon’s rank-sum test, and *P* values were adjusted for multiple comparisons using Bonferroni’s correction method. Ligands and receptors were considered significantly different if *P* was less than 0.05. The resulting data were visualized using Cytoscape (v3.9.1) software (https://cytoscape.org/) (38). The complete R script, including figure-specific visualization methods, is publicly available on GitHub (https://github.com/Galban-Lab).

### Histology, immunofluorescence, and quantification.

Mouse lungs from all experimental groups were perfused by injecting 10–20 mL of 1× PBS into the right ventricle of the heart until lungs turned white. Lungs were then sectioned into multiple pieces for downstream assays: RNA isolation, protein isolation, or histology. Right lung lobe sections were typically used for histology and fixed in 10% neutral buffered formalin and stored in 70% ethanol before embedding in paraffin. Sectioning, mounting, and H&E staining were performed by the University of Michigan Histology Core. Additional sections were used for coimmunofluorescence using the Alexa Fluor 488 Tyramide SuperBoost Kit (Invitrogen, B40943). Slides were deparaffinized and rehydrated and then washed in deionized water for 5 minutes. Antigen unmasking was performed by boiling slides in 1× CITRA or EDTA buffer for p-EGFR. Slides were quenched with 3% hydrogen peroxide for 15 minutes and then blocked with 20% goat serum. Primary antibody was left on overnight at 4°C in a humidified chamber. The following day, slides were incubated with poly-HRP anti-rabbit secondary antibody for 1 hour. Slides were washed and then incubated in tyramide signal amplification (TSA) solution for 10 minutes. Stop reagent was then added directly to the TSA solution and left for 5 minutes. Antigen unmasking was repeated with 1× CITRA and slides were blocked with 1% BSA for 30 minutes. Primary antibody was left on overnight in a humidified chamber at 4°C. The following day, slides were incubated with secondary antibody for 45 minutes. DAPI was added during the last 5 minutes, and then slides were mounted with Prolong Gold anti-fade reagent (Invitrogen, P36930). Antibody information is presented in Supplemental Table 1. Bright-field images (×10 or ×20) were acquired with an Olympus BX-53 microscope, an Olympus DP80 digital camera, and Olympus cellSens standard software. Immunofluorescence images were acquired with the Stellaris 8 Falcon Confocal Microscopy System using LAS X Software (Leica Microsystems). For quantification, at least 3 images were obtained per mouse and at least 3 mice were included in each experimental group. CellProfiler 4.2.6 (39) was used to quantify all immunofluorescence images. We quantified percentage positive area – PDGFR, F4/80, MPO – as all staining above a specified threshold over the total area of the image. Percentage colocalization – αSMA/PDGFR, MPO/F4/80, p-EGFR/ECAD – was quantified as area from stain 1 that overlapped with area from stain 2. For percentage positive cells – Ki67/ECAD, p-ERK/ECAD, CC3/ECAD, p-STAT3/PDGFR – we counted total nuclei and classified those that were positive for each individual stain as positive; all others were filtered out. Single-positive cells were related, and double-positive cells were classified. Double- and single-positive cells were counted and specified quantifications were performed. For each stain, images from the same mouse were averaged and plotted as a percentage. Ordinary 1-way ANOVA with Tukey’s post hoc test was performed to determine statistical significance between groups.

### Lung lesion counting.

To quantify the number of lesions per section of the lungs in all experimental groups, we scanned H&E-stained sections at ×1 magnification with the Nikon Supercool Scan 5000, as previously described (40). Three different readers counted lesions on at least 5 sections per experimental group. The average number of lesions was corrected by the size of the lung section (whole lung, half, quarter of the lung) per experimental group by dividing the number of lesions for whole or half lungs. Statistical significance was determined by ordinary 1-way ANOVA using GraphPad Prism. To quantify percentage tumor area, we imported slide scans into ImageJ (NIH), converted scans to 8-bit images, adjusted the image threshold to encompass the total lung area, and measured total lung area. Individual tumors were manually circled and their areas measured. The sum of all tumor areas was divided by total lung area and plotted as a percentage. Ordinary 1-way ANOVA with Tukey’s post hoc test was performed to determine statistical significance between groups.

### qRT-PCR.

Total RNA was extracted using the RNeasy Mini Kit (Qiagen, 74104). Purified total RNA samples were quantified using a spectrophotometer (NanoDrop ND-1000, NanoDrop Technologies). cDNA was synthesized using random hexamers and oligo-dT primers (QuantiTect Reverse Transcription Kit, Qiagen) in a 2720 Thermal Cycler (Bio-Rad). cDNA samples for qRT-PCR were prepared using 1× Fast-SYBR Green PCR Master Mix (Applied Biosystems). Primers are listed in Supplemental Table 2. qRT-PCR was performed in technical and biological triplicates in a QuantStudio3 thermal cycler (Applied Biosystems). Mouse and human peptidylprolyl isomerase A and β-actin were used as housekeeping controls. Data were analyzed through relative quantification with the –ΔΔCt method.

### Western blotting.

Tissues were homogenized and lysed with RIPA lysis buffer (Thermo Fisher Scientific) supplemented with protease inhibitors (Complete Protease Inhibitor Cocktail, Roche, 4693116001) and phosphatase inhibitors (PhosSTOP, Roche). Similarly, cells were lysed with RIPA lysis buffer (Thermo Fisher Scientific) supplemented with protease inhibitors (Complete Protease Inhibitor Cocktail, Roche) and phosphatase inhibitors (PhosSTOP, Roche). We determined protein concentrations of whole-cell lysates using Lowry assays (Bio-Rad). Twenty micrograms of total protein was prepared in LDS sample buffer (Invitrogen), separated in denaturing Bis-Tris gels (Invitrogen), and transferred to nitrocellulose membranes (GE Healthcare). Membranes were blocked in 5% milk in 0.1% Tween 20/Tris-buffered saline (TBST) and incubated with primary antibodies against p-p44/42 MAPK (p-ERK1/2; Thr202/Tyr204), total ERK, RAS^G12D^ (D8H7), and p53 in TBST overnight at 4°C, or with HRP-conjugated β-actin for 1 hour at room temperature. Manufacturer details for antibodies are listed in Supplemental Table 1. Secondary antibodies were purchased from Jackson ImmunoResearch. ECL-Plus substrate (Bio-Rad) and the Bio-Rad ChemiDoc MP imager were used according to the manufacturer’s recommendations.

### Cell lines.

We established primary mouse lung cancer cell lines LC3-547 and LC3-545, derived from the *Ccsp-rtTa; TetO-Kras^G12D^; Trp53^LSL-R172H/+^* mouse model, from lung tissue of GEMM mice treated with dox and ad-Cre. In both murine cell lines, oncogenic KRAS expression can be induced or inactivated by addition of 1 μg/mL of doxycycline hyclate (Sigma-Aldrich, D9891) to medium or withdrawn by replacing with media excluding dox. Cells were used at low passage and genotyped for the *Ccsp-rtTa*, *TetO-Kras^G12D^*, and *Trp53^LSL-R172H/+^* transgenes. Cells tested negative for mycoplasma. We isolated NLF-2522 from normal lung tissue of control mice. Both cell lines were cultured in DMEM (Gibco) with 10% heat-inactivated FBS (HI-FBS; Gibco) and 1% Pen/Strep (Gibco). Murine fibroblast cells (NLF-2522) were not transformed or immortalized, but likely spontaneously immortalized.

The KPL-86 primary mouse lung cancer cell line was provided by David DeNardo (Washington University School of Medicine, St. Louis, Missouri, USA) (21). Briefly, the KPL-86 line was generated from lung tissues of 9-month-old *Kras^LSL-G12D^*; *Trp53^fl/+^* mice treated with ad-Cre recombinase. KPL-86 cells were maintained in collagen-coated tissue culture dishes in DMEM/F12 (Gibco) with 10% HI-FBS (Gibco) and 1% Pen/Strep (Gibco). We obtained human lung adenocarcinoma A-549 (KRAS^G12S^) and A-427 (KRAS^G12D^) cell lines from ATCC (CCL-185 and CRL-1642, respectively). Cells were maintained in RPMI with 10% HI-FBS and 1% Pen/Strep. The human fibroblast CCL-210 cell line was provided by Marc Peters-Golden (University of Michigan). The CCL-210 (CCD-19Lu) fibroblast cell line originally from ATCC was not further transformed or immortalized in our lab. The CCL-210 line was cultured in low-glucose DMEM (Gibco) with 10% HI-FBS, 1× GlutaMAX-I Supplement (Gibco, A1286001), and 1× Antibiotic-Antimycotic (Gibco). All cells were cultured at 37°C with 5% CO_2._ We performed monthly mycoplasma testing with the MycoAlert Mycoplasma Detection Kit (Lonza, LT07-318).

### In vitro experiments using CM.

TCM was generated from murine and human KRAS^G12D^-expressing cells LC3-547 and A427, respectively. LC3-547 cells were cultured in complete DMEM (1% HI-FBS and 1% Pen/Strep) containing dox (1 μg/mL) and A427 cells in complete RPMI. We treated cells with MRTX or AMG 510 (Soto) at concentrations indicated in the figure legends or with equimolar concentration of DMSO. Treatment was repeated daily. Media were collected 48 hours after seeding and centrifuged at 300*g* for 10 minutes at 4°C. Supernatant was collected and stored at −80°C. We used TCM for multiplex ELISA (Luminex analysis, see below) or conditioning fibroblasts. Murine and human fibroblasts NFL-2522 and CCL-210, respectively, were incubated with TCM for 24 hours prior to collecting CAF-conditioned media (CAF CM) or fibroblasts themselves for Luminex analysis or qRT-PCR.

### Luminex assays.

Multiplex ELISA (Luminex) assays were performed on TCM and CAF CM. In brief, TCM or CAF CM was thawed and 200 μL were submitted to the Chemistry Laboratory of the Michigan Diabetes Research Center (MDRC), where analytes were measured using the MILLIPLEX Mouse Cytokine/Chemokine Magnetic Bead Panel (Millipore, MCYTMAG-70K-PX32) for murine samples and Human Cytokine/Chemokine/Growth Factor Panel A (Millipore, HCYTA-60K-PX48) for human samples. Standard curves were generated using non-conditioned media and analytes were quantified on the Luminex 200 instrument.

### Bone marrow isolation and macrophage polarization.

TCM or FB CM were used for the macrophage polarization assay. For macrophage polarization, we isolated bone marrow tissue from femurs and tibia of control transgenic mice (single transgenics or wild type; not treated with dox or ad-Cre) by carefully flushing out the marrow until the bone was white, which indicates that all marrow has been removed. Cells were centrifuged at 433*g* for 7 minutes at 4°C and the pellet was incubated with 1 mL red blood cell lysis buffer. Once isolated, 2 × 10^6^ BMDMs were cultured directly with CM for 6 days. Fresh CM were added on the third day. Through this process, BMDMs differentiate and polarize to TAMs. We treated BMDMs from the ON plus CXCR inhibitor group with 10 nM CXCR2 inhibitor SB225002 (Selleckchem, S7651). After 6 days, TAMs were harvested and lysed to extract total RNA.

### Statistics.

Differential expression analysis at the single-cell level was performed using the FindMarkers function from the Seurat R package. A nonparametric Wilcoxon’s rank-sum test was used to determine *P* values, and *P* values were corrected using Bonferroni’s correction. Adjusted *P* values are reported, with a *P* value of less than 0.05 indicating a significant difference.

GSEA was performed using the fgsea R package. This package computes a running sum statistic that estimates the association of the gene set with the phenotype under study. A *P* value associated with this statistic was also computed using an adaptive multilevel split Monte Carlo simulation. *P* values were then corrected using the Benjamini and Hochberg method.

Comparison of survival curves was analyzed using a log-rank (Mantel-Cox) test. The *P* value is reported. Median survival was analyzed using a 1-way ANOVA with Tukey’s post hoc test, which gives a multiplicity-adjusted *P* value for each comparison. This adjusted *P* value is reported.

To quantify percentage tumor area on H&E-stained histological lung sections, we imported slide scans into ImageJ, converted scans to 8-bit images, adjusted the image threshold to encompass the total lung area, and measured total lung area. Individual tumors were manually circled, and their areas measured. The sum of all tumor areas was divided by total lung area and plotted as a percentage. Ordinary 1-way ANOVA with Tukey’s post hoc test was performed to determine statistical significance between groups. This gives a multiplicity-adjusted *P* value for each comparison, which is reported.

To quantify the number of lesions per section of the H&E-stained lungs in all experimental groups, 3 different readers counted lesions on at least 5 sections per experimental group. The average number of lesions was corrected by the size of the lung section (whole lung, half, quarter of the lung) per experimental group by dividing the number of lesions for whole or half lungs. Ordinary 1-way ANOVA with Tukey’s post hoc test was performed to determine statistical significance between groups. This gives a multiplicity-adjusted *P* value for each comparison, which is reported.

For quantification of the immunofluorescently stained lungs in all experimental groups, at least 3 images were taken per mouse and at least 3 mice were included in each experimental group. CellProfiler 4.2.6 (39) was used to quantify all immunofluorescence images. We quantified percentage positive as all staining above a specified threshold over the total area of the image. Percentage colocalization was quantified as the area from stain 1 that overlapped with area from stain 2. For percentage positive cells, we counted total nuclei and classified those that were positive for each individual stain as positive; all others were filtered out. Single-positive cells were related, and double-positive cells were classified. Double- and single-positive cells were counted and specified quantifications were performed. For each stain, images from the same mouse were averaged and plotted as a percentage. Ordinary 1-way ANOVA with Tukey’s post hoc test was performed to determine statistical significance between groups. This gives a multiplicity-adjusted *P* value for each comparison, which is reported.

For qRT-PCR data analyses, results were analyzed by relative quantification with the –ΔΔCt method. For comparisons with only 2 groups, statistical significance was determined with a 2-tailed Student’s *t* test for unpaired samples. A *P* value less than 0.05 was considered statistically significant. For comparisons with more than 2 groups, statistical significance was determined using 1-way ANOVA with Tukey’s post hoc test. This gives a multiplicity-adjusted *P* value for each comparison, which is reported.

### Study approval.

All animal studies were conducted in accordance with the University of Michigan’s Institutional Animal Care and Use Committee–approved protocol PRO00010349.

### Data availability.

The datasets supporting the current study are available from the corresponding author (SG). Raw data are also provided in the Supporting Data Values file for this study. Raw and processed RNA data are available through the NCBI GEO database under accession number GSE281744. The complete R script, including figure-specific visualization methods, is publicly available on GitHub (https://github.com/Galban-Lab). Material or data that require a Material Transfer Agreement (MTA) can be provided by the University of Michigan pending scientific review and the execution of an MTA negotiated by the university’s Office of Technology Transfer. Requests for data that require an MTA should be submitted to the corresponding author (SG).

## Author contributions

MPM and SG conceptualized and supervised all aspects of the study. They provided all resources and secured funding. TLF, MPM, and SG developed methods and supervised studies. ELLO, IB, EM, JML, SFF, CEE, RH, SC, MR, LR, KB, KYA, CJG, TLF, and YZ conducted experiments. All work using software was conducted by ELLO and IB. ELLO, IB, TLF, MPM, and SG curated data. ELLO, IB, EM, CEE, SC, and LR analyzed results. ELLO, IB, SC, and LR validated results. ELLO, IB, MPM, and SG wrote the manuscript. ELLO, IB, TLF, MPM, and SG reviewed and edited all drafts. ELLO and IB contributed equally as co–first authors to the research for this article. ELLO is listed first because she had a greater role in preparing the manuscript for submission.

## Supplementary Material

Supplemental data

Unedited blot and gel images

Supporting data values

## Version 1 01/09/2025

Electronic publication
