# Supplementary material for: KRASG12D drives immunosuppression in lung adenocarcinoma through paracrine signaling
Source: JCI Insight. 2025 Jan 9;10(1):e182228. doi: 10.1172/jci.insight.182228 (PMC11721295; doi:10.1172/jci.insight.182228)
Supplement: Supplemental data [file jciinsight-10-182228-s174.pdf]

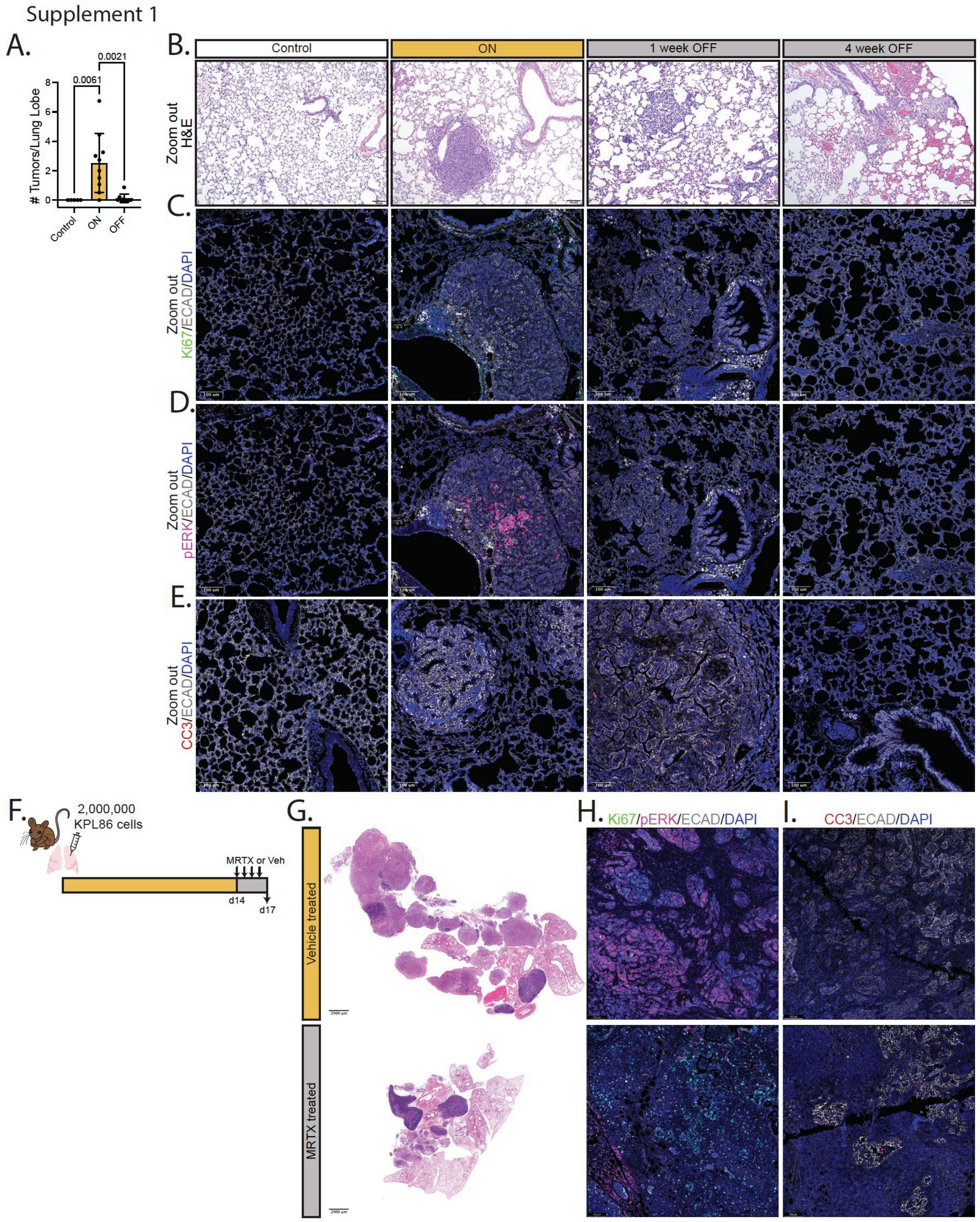

Supplemental Figure 1: (A) Quantification of lesions in H&E-stained lung sections from L-iKras and control mice with (1, 2 and 4 weeks) and without dox withdrawal (17-25 weeks). Data represent mean ± SEM. Statistical analysis was performed with one-way ANOVA with post-hoc Tukey multiple comparison test. A *P* value less than 0.05 was considered statistically significant. **(B)** Zoomed out representative images of H&E from Figure 1E. Scale bar = 100 µm. **(C)** Zoomed out representative images of Ki67/E-cadherin/DAPI from Figure 1G. Scale bar = 100 µm. **(D)** Zoomed out representative images of pERK/E-cadherin/DAPI from Figure 1I. Scale bar = 100 µm. **(E)** Zoomed out representative images of CC3/E-cadherin/DAPI from Figure 1K. Scale bar = 100 µm. **(F)** Scheme depicting orthotopic injection of 200,000 KPL86 cells followed by twice daily intraperitoneal injection (ip) of MRTX1133 (MRTX) or vehicle for 2 days. **(G)** Slide scans of lung H&E stains from the orthotopic KPL lung model treated with vehicle or MRTX. Scale bar = 2000 µm. **(H)** Representative images of Ki67/pERK/E-cadherin/DAPI from the orthotopic KPL lung model treated with vehicle or MRTX. Scale bar = 100µm. **(I)** Representative images of CC3/E-cadherin/DAPI from the orthotopic KPL lung model treated with vehicle or MRTX. Scale bar = 100 µm.

Supplement 2

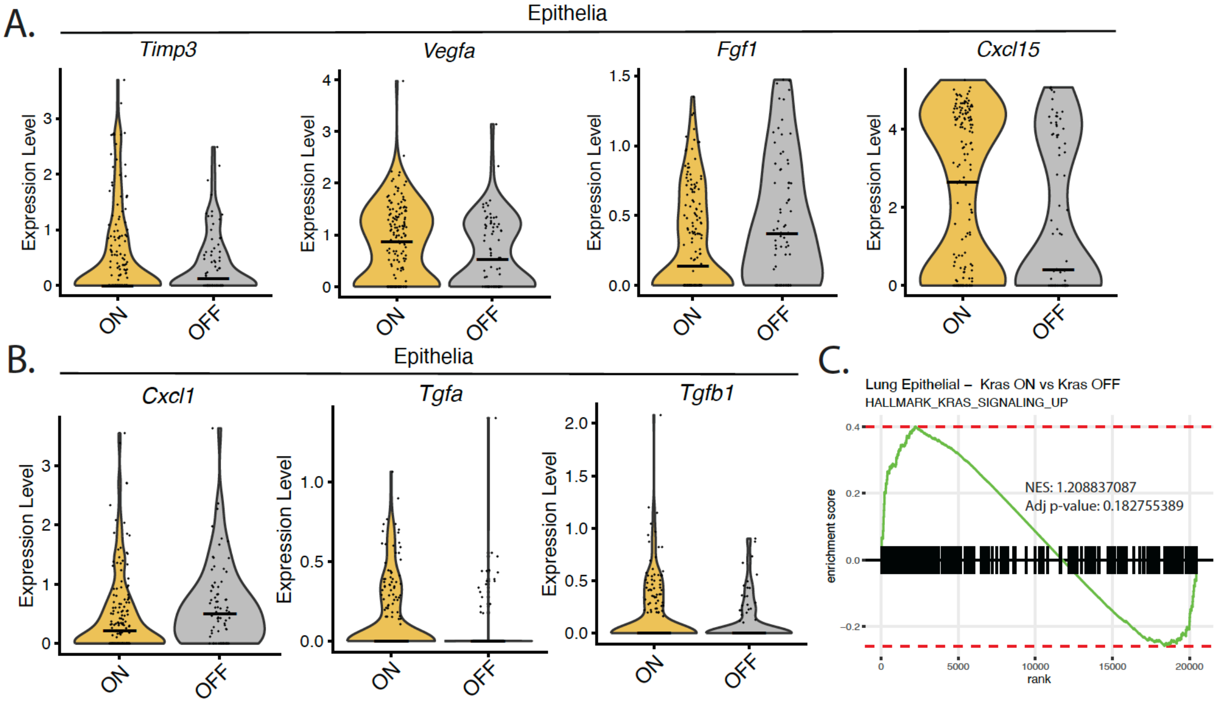

Supplemental Figure 2: (A) Violin plots of *Timp3*, *Vegfa*, *Fgf1*, and *Cxcl15* comparing expression levels between total epithelial cells from Kras ON and Kras OFF samples. Median expression is marked. **(B)** Violin plot of *Cxcl1, Tgfa,* and *Tgfb1* comparing expression levels between total epithelial cells from Kras ON and Kras OFF samples. Median expression is marked. **(C)** GSEA plot of Kras ON vs Kras OFF lung epithelia showing the running enrichment score for the 'HALLMARK_KRAS_SIGNALING_UP' gene set. NES = 1.208837087. Adjusted *P* value = 0.182755389.

Supplement Figure 3

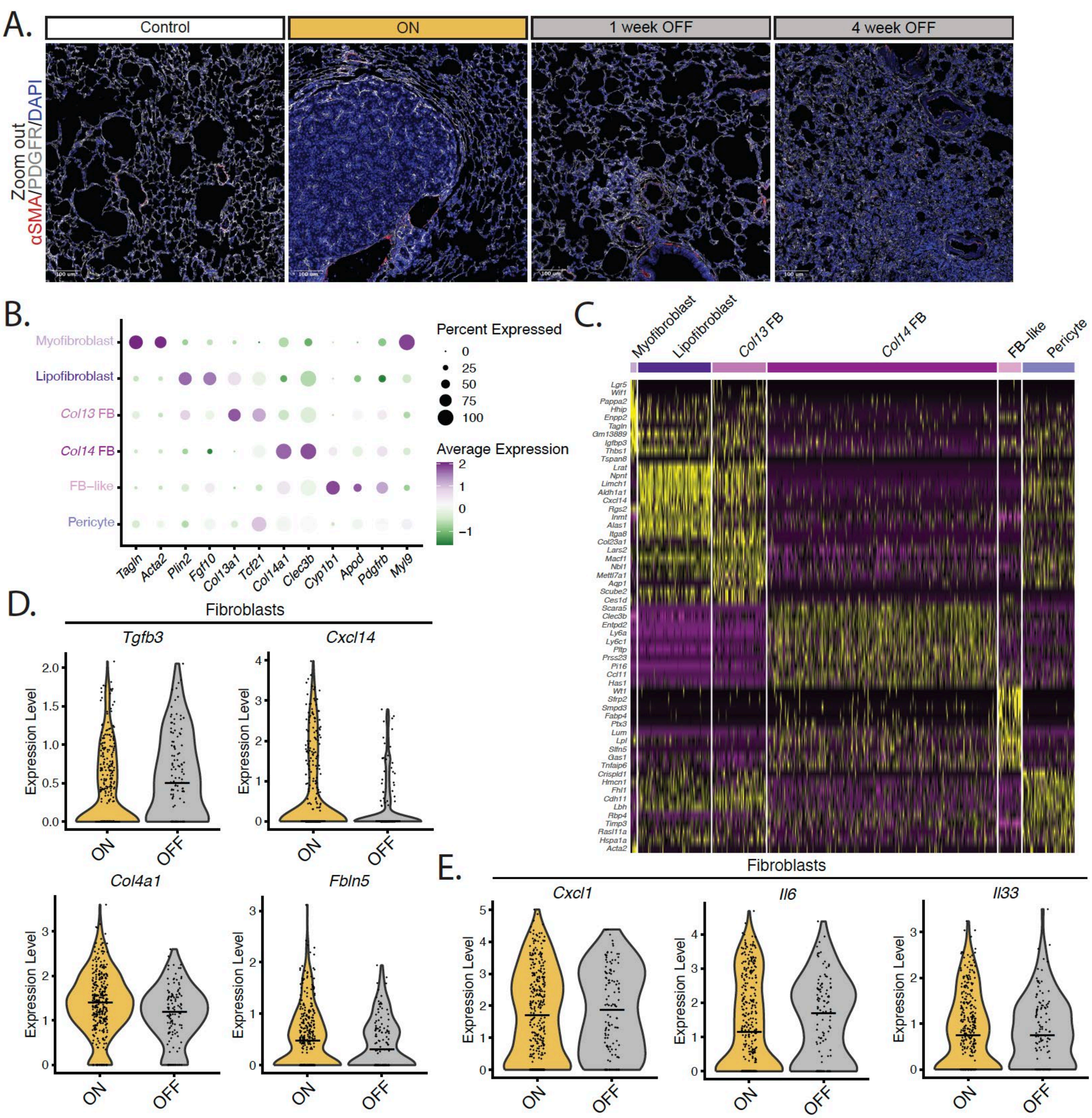

Supplemental Figure 3: (A) Zoomed out representative images of αSMA/PDGFR/DAPI from Figure 3A. Scale bar = 100 μM. **(B)** Dot plot of markers used to identify different types of fibroblasts. Dot size shows expression frequency, dot color shows average expression. **(C)** Heatmap of top differentially expressed genes in the fibroblast subclusters identified from Kras ON (21 weeks ON) vs Kras OFF (20 weeks ON + 1 week OFF) lung. **(D)** Violin plots of *Tgfb3*, *Cxcl14*, *Col4a1*, and *Fbln5* comparing expression levels between fibroblasts from Kras ON and Kras OFF samples. Median expression is marked. **(E)** Violin plots of *Cxcl1*, *Il6*, and *Il33* comparing expression levels between fibroblasts from Kras ON and Kras OFF samples. Median expression is marked.

Supplemental Figure 4

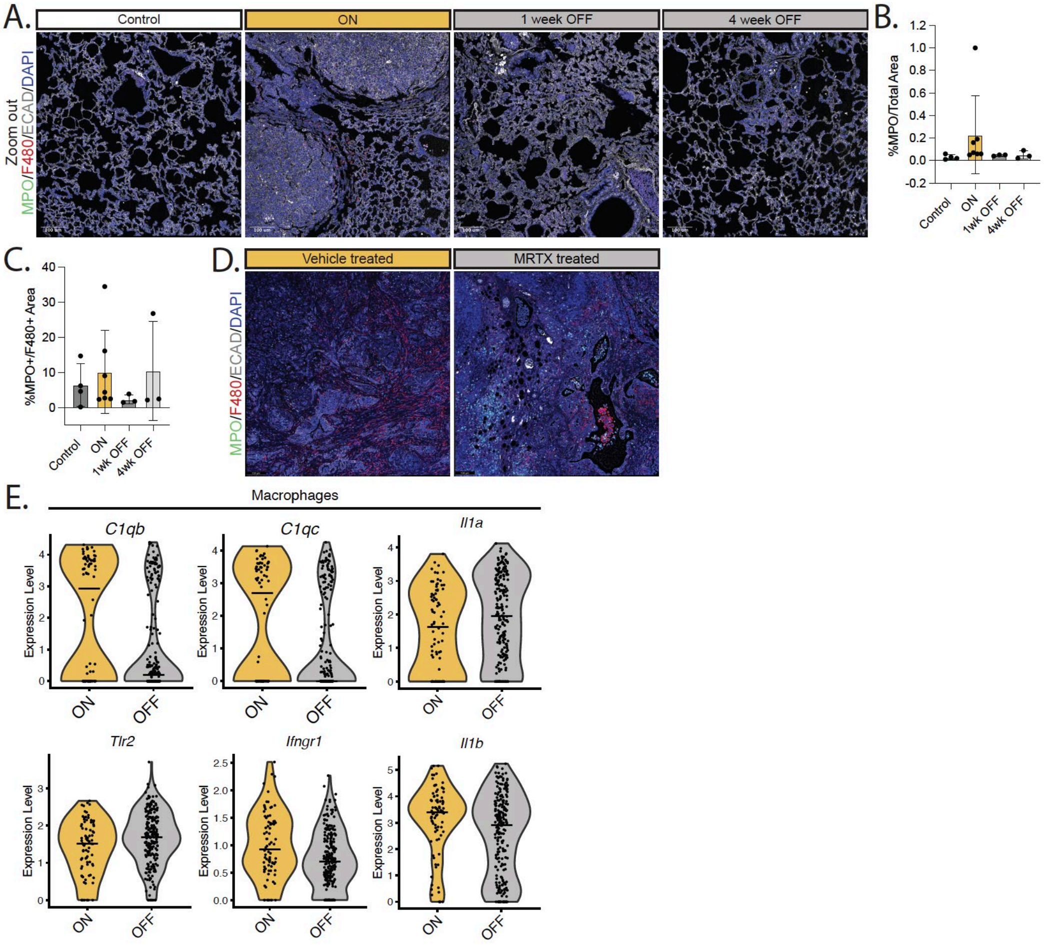

Supplemental Figure 4: (A) Zoomed out representative images of MPO/F4/80/ECAD/DAPI from Figure 4A. Scale bar = 100 µM. **(B)** Quantification of percent MPO+ area of total area. **(C)** Quantification of percent MPO+ area of total F4/80+ area. **(D)** Representative images of MPO/F4/80/E-cadherin/DAPI from the orthotopic KPL lung model treated with vehicle or MRTX. Scale bar = 100 µm. **(E)** Violin plots of *C1qb*, *C1qc*, *Il1a*, *Tlr2*, *Ifngr1*, and *Il1b* comparing expression levels between macrophages from Kras ON and Kras OFF samples. Median expression is marked.

Supplemental Figure 5

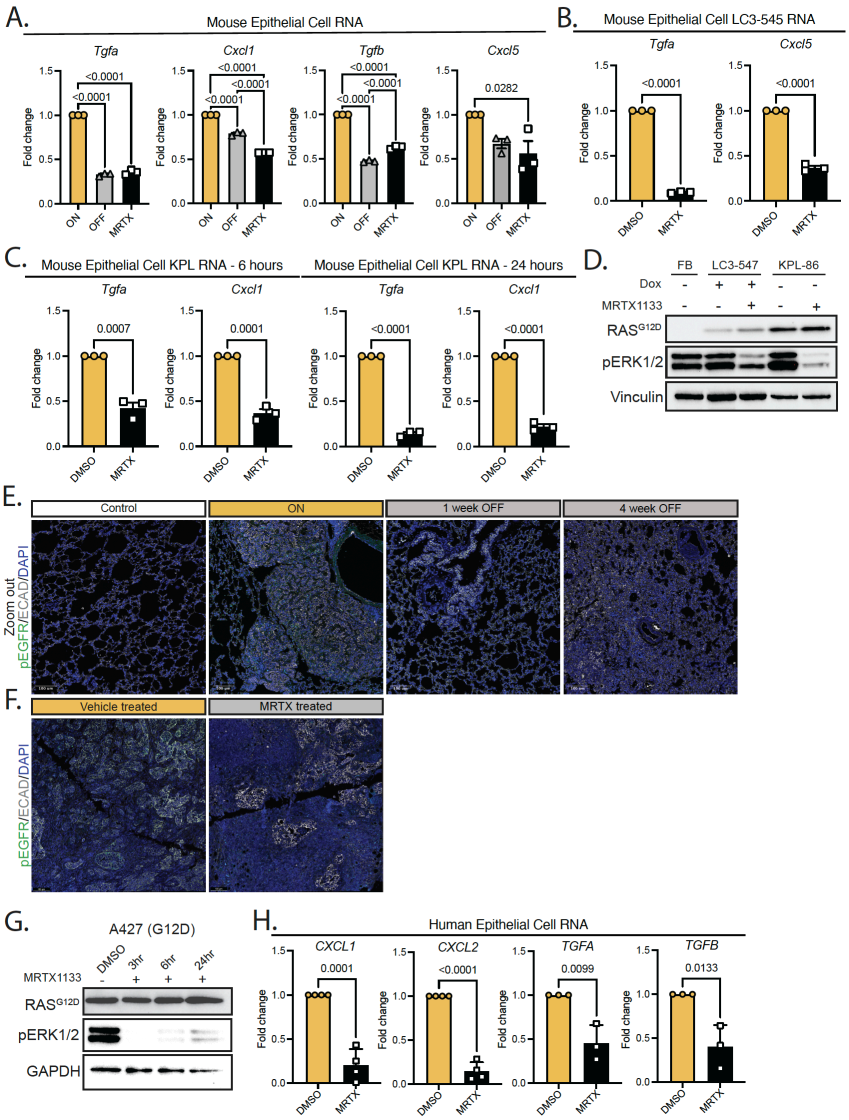

Supplemental Figure 5: (A) qRT-PCR for *Tgfa, Cxcl1, Tgfb* and *Cxcl5* expression in LC3-547 cells grown in media containing doxycycline, with and without 500 nM MRTX, or in media where doxycycline has been removed for 24 hours. **(B)** qRT-PCR for *Tgfa* and *Cxcl5* expression in LC3-545 cells treated with DMSO or 500 nM MRTX for 6 hours. Data represented ± SEM and statistical significance was determined with two-tailed Student's t-test for unpaired samples. A *P* value less than 0.05 was considered statistically significant. **(C)** qRT-PCR for *Tgfa* and *Cxcl1* expression in KPL cells treated with DMSO or 500 nM MRTX for 6 or 24 hours. Data represented ± SEM and statistical significance was determined with two-tailed Student's t-test for unpaired samples. A *P* value less than 0.05 was considered statistically significant. **(D)** Western blot for indicated proteins performed on lysates from LC3-547 (L-iKras model) and KPL-86 (KP model) cells treated with MRTX or equimolar concentrations of DMSO. Murine fibroblast lysates were used as negative controls for $Kras^{G12D}$ expression. **(E)** Zoomed out representative images of pEGFR/ECAD/DAPI from Figure 5H. Scale bar = 100 µM**.** **(F)** Representative images of pEGFR/ECAD/DAPI staining on lung sections from the orthotopic KPL lung model treated with vehicle or MRTX twice daily at 30 mg/kg, ip, for two days. **(G)** Western blot of Ras^{G12D} expression in human $Kras^{G12D}$ lung adenocarcinoma cell line, A427, upon treatment with MRTX at indicated time points. **(H)** qRT-PCR for *CXCL1, CXCL2, TGFA,* and *TGFB* expression in cells treated with DMSO or 500 nM MRTX for 6 hours. Data represented ± SEM. Statistical significance was determined with two-tailed Student's t-test for unpaired samples. A *P* value less than 0.05 was considered statistically significant.

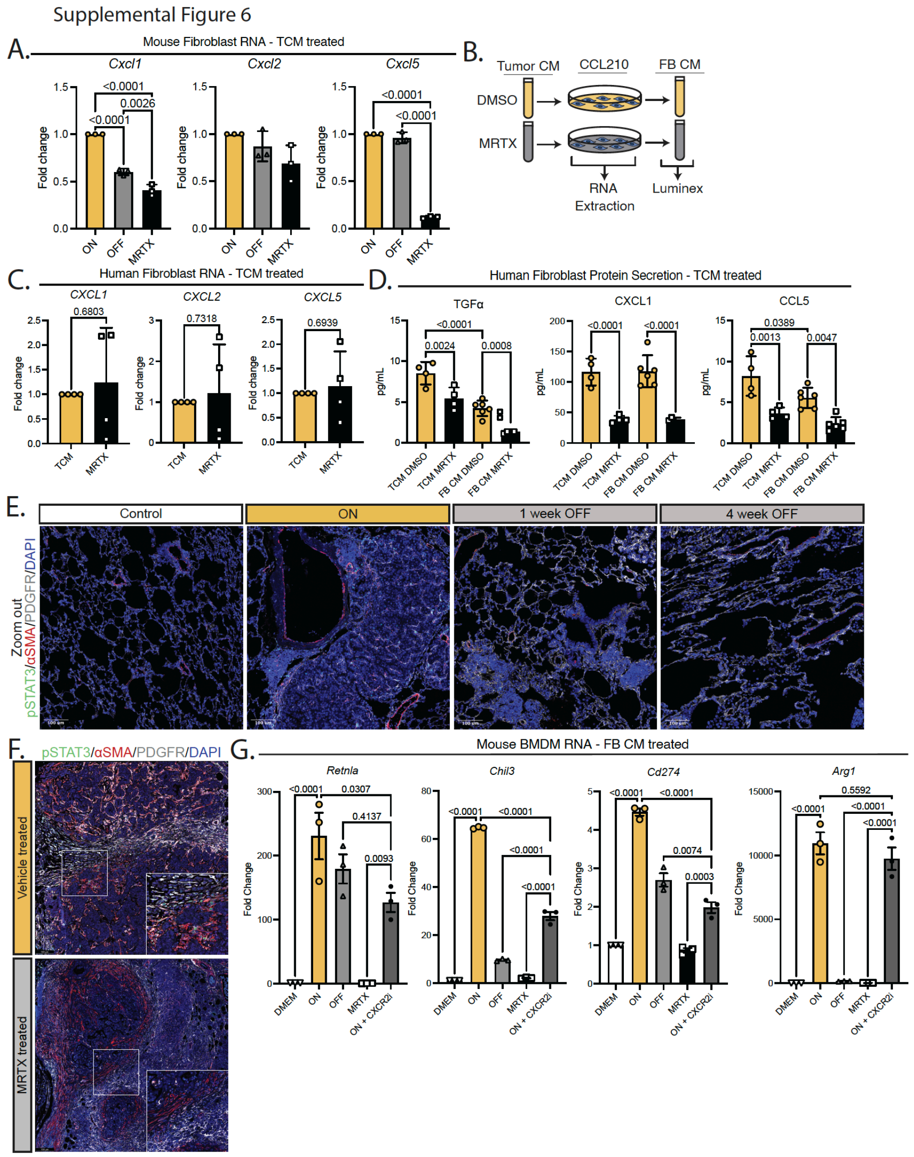

Supplemental Figure 6: (A) NLF-2522 fibroblasts treated with TCM from LC3-547 cells cultured with (ON) or without (OFF) doxycycline and treated with MRTX or equimolar concentrations of DMSO (only for doxycycline treated cells) and expression of *Cxcl1, Cxcl2,* and *Cxcl5* assessed by qRT-PCR. **(B)** Experimental outline indicating that adult human lung fibroblast cells, CCL210, were cultured in TCM from A427 cells treated with 100 nM MRTX or equimolar DMSO for 24 hours before harvesting RNA and conditioned media (fibroblast CM). All experiments were repeated at least three times, each time with three technical replicas. **(C)** CCL210 fibroblasts treated with TCM from DMSO and MRTX treated A427 cells and expression of *CXCL1, CXCL2,* and *CXCL5* assessed by qRT-PCR. **(D)** Quantification of CXCL1 and CCL5 cytokine secretion of human fibroblasts treated with TCM using multiplex ELISAs. Data represented ± SEM and statistical significance determined with two-tailed Student's t-test for unpaired samples. A *P* value less than 0.05 was considered statistically significant. **(E)** Zoomed out representative images of pSTAT3/αSMA/PDGFR/DAPI from Figure 6E. Scale bar = 100 μM. **(F)** Representative images for pSTAT3/αSMA/PDGFR/DAPI staining on lung sections from the orthotopic KPL-86 lung cancer model where mice were treated with vehicle or MRTX for two days twice daily at 30 mg/kg ip. **(G)** Quantitative reverse-transcription polymerase chain reaction for M2 markers: *Retnla, Chil3, Cd274,* and *Arg1* expression in treated BMDMs from ON, OFF, MRTX, and DMEM treated cells. A fifth experimental group included addition of CXCR2 inhibitor to the ON media condition. Data represented ± SEM. Statistical analysis was performed with one-way ANOVA with post hoc Tukey HSD test. A *P* value less than 0.05 was considered statistically significant.

Supplemental Figure 7

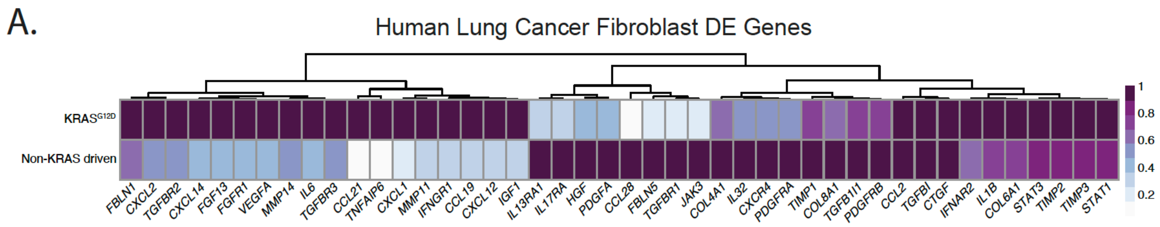

Supplemental Figure 7: (A) Heatmap showing averaged scRNAseq expression data (relative to the highest expressor) for genes in human fibroblasts (Maroni et al., *Commun Biol* 2021, PMID 33854168) curated from the differential gene expression list.

Supplemental Tables 1 and 2

Supplemental Table S1: Immunohistochemistry, Western Blot Antibodies.

**Antibody**	**Supplier**	**Catalog number**
Ki67	Abcam	Ab15580
p-ERK (T202/Y204)	Cell Signaling	4370
CC3	Cell Signaling	9661
αSMA	Sigma Aldrich	A2547
PDGFR	Abcam	Ab32570
Myeloperoxidase	Cell Signaling	15178
F4/80	Cell Signaling	70076
ECAD	Cell Signaling	14472
pEGFR (Tyr1068)	Cell Signaling	3777
pSTAT3 (Tyr705)	Cell Signaling	9145
Ras (G12D mutant specific) (D8H7)	Cell Signaling	14429
Total Ras (D2C1)	Cell Signaling	8955
Anti-β-Actin-HRP	Abcam	Ab8226
GAPDH-HRP	Abcam	Ab9482
Vinculin (E1E9V) XP	Cell Signaling	13901

Supplemental Table S2: Primers utilized for qRT-PCR. Ms = murine, Hs = human.

Oligo Name	Sequence (5'-3)
ms Cxcl1 fwd	CTGGGATTCACCTCAAGAACATC
ms Cxcl1 rev	CAGGGTCAAGGCAAGCCTC
Hs CXCL1 fwd	AGCTTGCCTCAATCCTGCATCC
Hs CXCL1 rev	TCCTTCAGGAACAGCCACCAGT
ms Cxcl2 fwd	CCAACCACCAGGCTACAGG
ms Cxcl2 rev	GCGTCACACTCAAGCTCTG
Hs CXCL2 fwd	GGCAGAAAGCTTGTCTCAACCC
Hs CXCL2 rev	CTCCTTCAGGAACAGCCACCAA
ms Cxcl5 fwd	TCCAGCTCGCCATTCATGC
ms Cxcl5 rev	TTGCGGCTATGACTGAGGAAG
Hs CXCL5 fwd	CAGACCACGCAAGGAGTTCATC
Hs CXCL5 rev	TTCCTTCCCGTTCTTCAGGGAG
ms Actb fwd	CATTGCTGACAGGATGCAGAAGG
ms Actb rev	TGCTGGAAGGTGGACAGTGAGG
Hs ACTB fwd	CACCATTGGCAATGAGCGGTTC
Hs ACTB rev	AGGTCTTTGCGGATGTCCACGT
ms Cyclophilin fwd	TCACAGAATTATTCCAGGATTCATG
ms Cyclophilin rev	TGCCGCCAGTGCCATT
Hs Cyclophilin fwd	CCCACCGTGTTCTTCGACATT
Hs Cyclophilin rev	GGACCCGTATGCTTTAGGATGA
ms Ppia fwd	TCACAGAATTATTCCAGGATTCATG
ms Ppia rev	TGCCGCCAGTGCCATT
Hs PPIA fwd	CCCACCGTGTTCTTCGACATT
Hs PPIA rev	GGACCCGTATGCTTTAGGATGA
ms PD-L1 (CD274) fwd	TGCGGACTACAAGCGAATCACG
ms PD-L1 (CD274) rev	CTCAGCTTCTGGATAACCCTCG
Hs PD-L1 (CD274) fwd	TGCCGACTACAAGCGAATTACTG
Hs PD-L1 (CD274) rev	CTGCTTGTCCAGATGACTTCGG
ms Tgfα fwd	CAGGCTCTGGAGAACAGCACAT
ms Tgfα rev	GACACATGCTGGCTTCTCTTCC
Hs TGFα rev	GAGCCCTCGGTAAGTATGTTTAG
Hs TGFα fwd	CATAGTGGAGGTGACTTGTTAGAG
ms Tgf β1 fwd	TGATACGCCTGAGTGGCTGTCT
ms Tgf β1 rev	CACAAGAGCAGTGAGCGCTGAA
Hs TGFβ1 fwd	GTTCAAGCAGAGTACACACAGC
Hs TGFβ1 rev	GTATTTCTGGTACAGCTCCACG
ms Arg1 fwd	AAAGGCCGATTCACCTGAGC
ms Arg1 rev	CTGAAAGGAGCCCTGTCTTGTA
ms Ccl5 fwd	GCTGCTTTGCCTACCTCTCC
ms Ccl5 rev	TCGAGTGACAAACACGACTGC
ms Csf2 fwd	ACTTTCTGCTTGTCATCCCC
ms Csf2 rev	CCATCCTGAGTTTCTAGCTCTTG
