# Supplementary material for: KRASG12D drives immunosuppression in lung adenocarcinoma through paracrine signaling
Source: JCI Insight. 2025 Jan 9;10(1):e182228. doi: 10.1172/jci.insight.182228 (PMC11721295; doi:10.1172/jci.insight.182228)

Raw data for Figure 1D

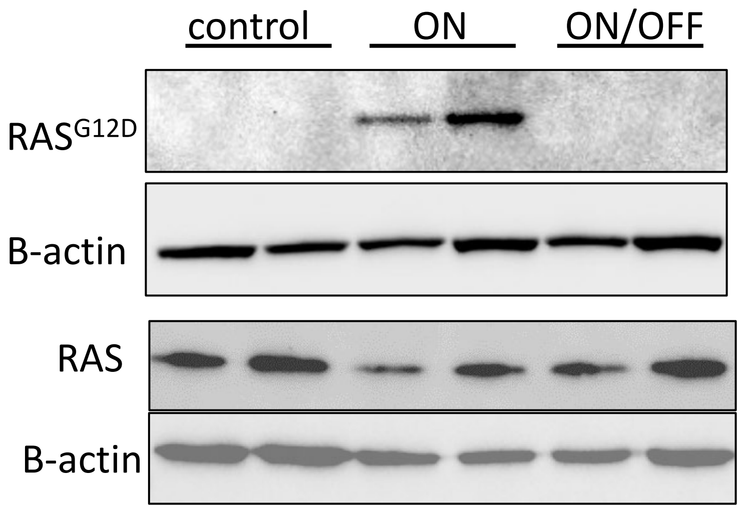

Raw data for Figure 1D

IB#80

1st antibody

Control KRAS On KRAS Off KRAS On/Off/On

Marker See Blue
LC5 Lung Tissue 1023
LC5 Lung Tissue 956
LC5 Lung Tissue 817
LC5 Lung Tissue 849
LC5 Lung Tissue 1049
LC5 Lung Tissue 1015
LC5 Lung Tissue 1013
LC5 Lung Tissue 1018

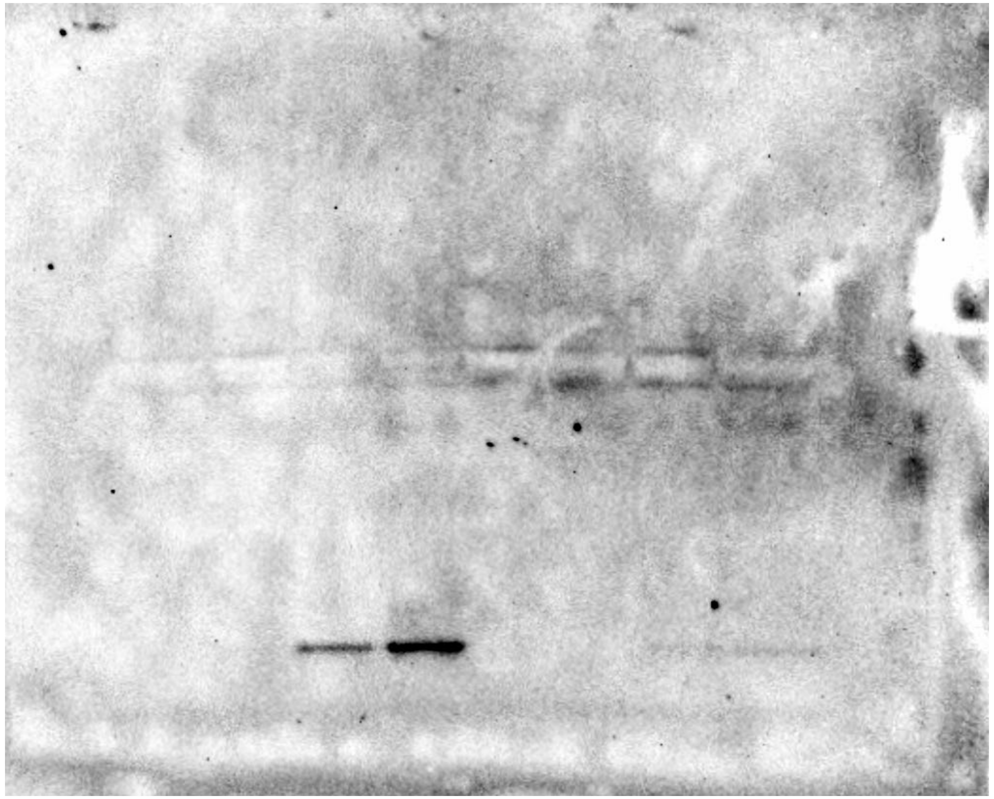

17 kDa RAS G12D

Composite image

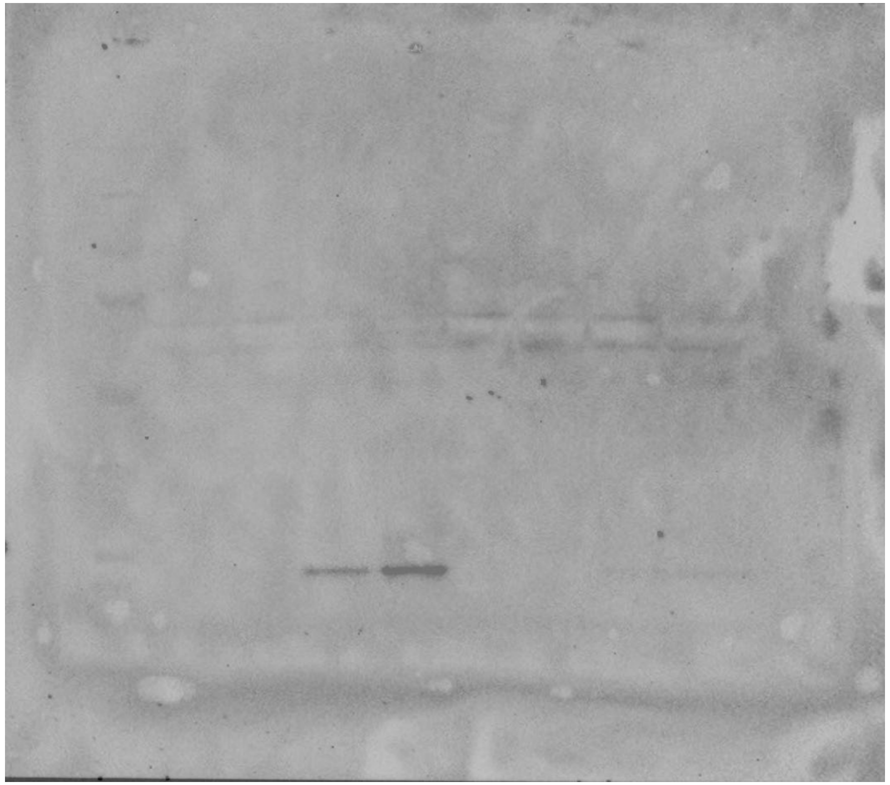

Raw data for Figure 1D

IB#80

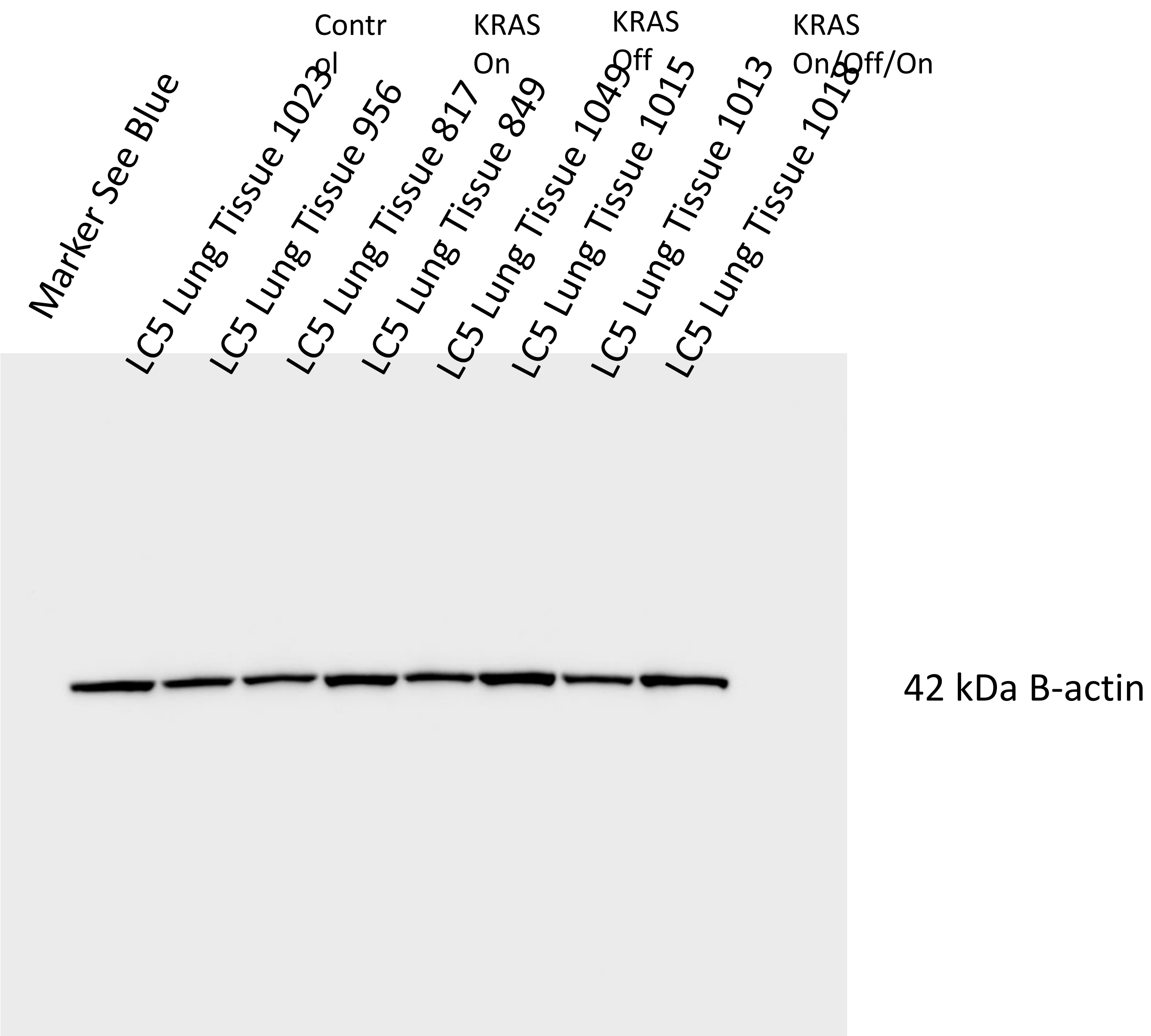

Raw data for Figure 1D

IB#80

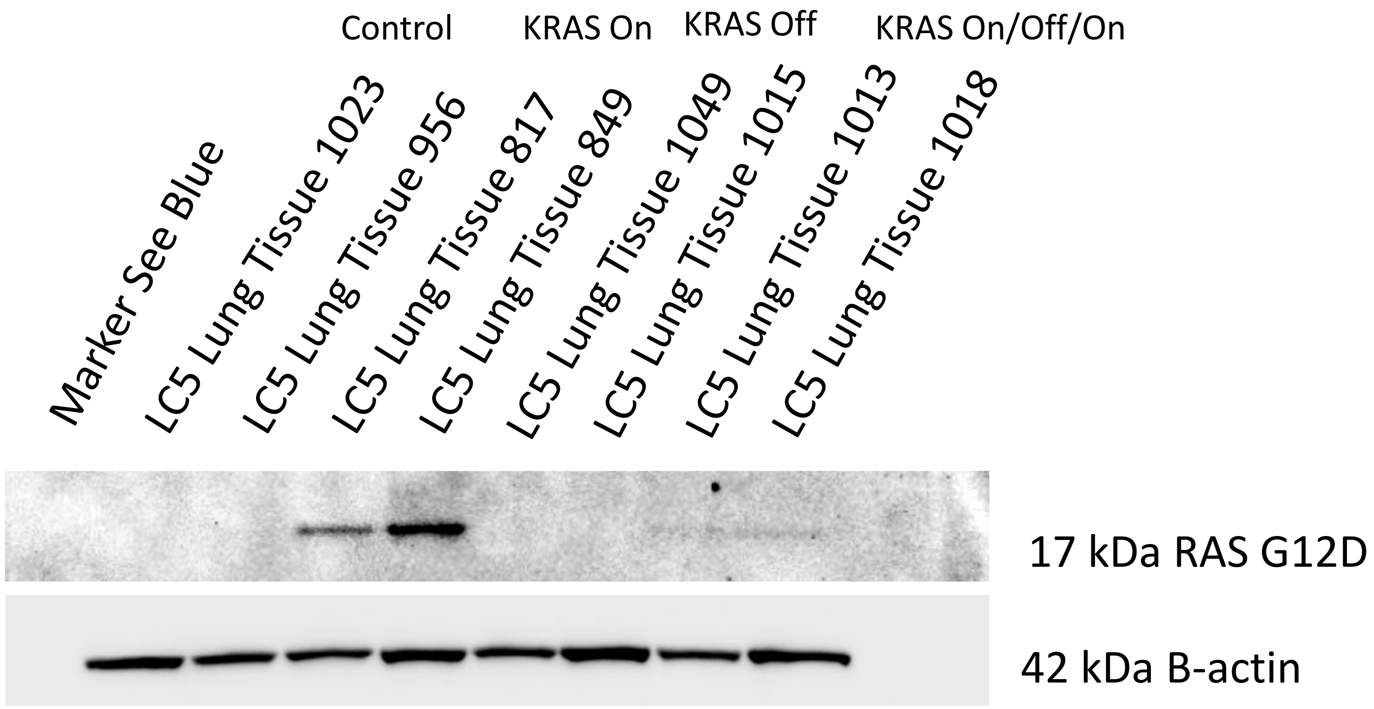

IB#80

Raw data for Figure 1D

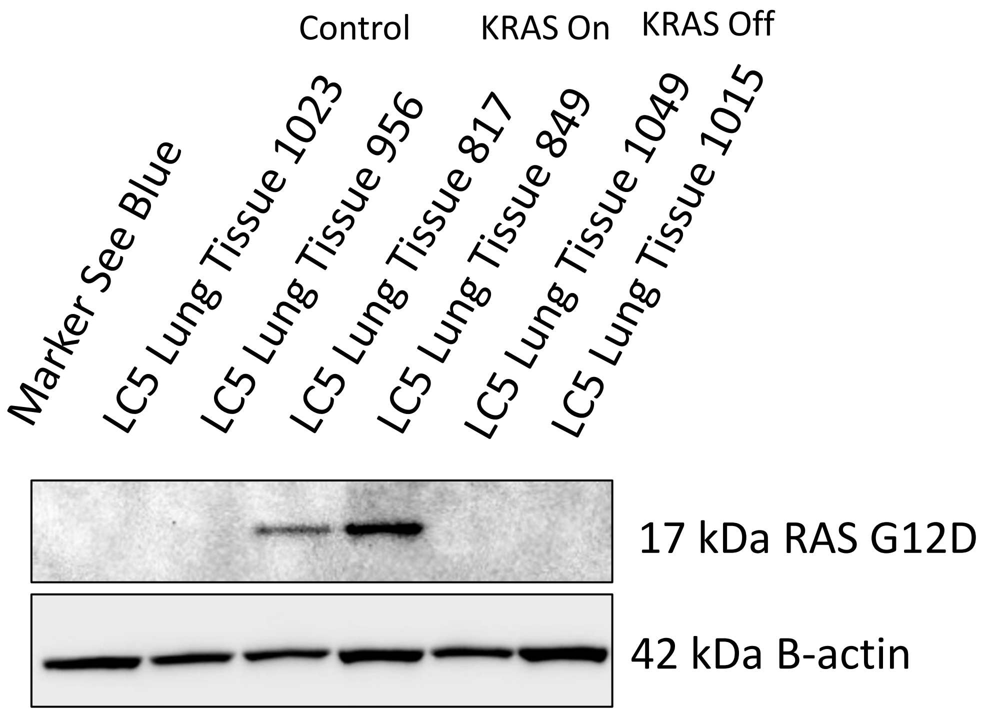

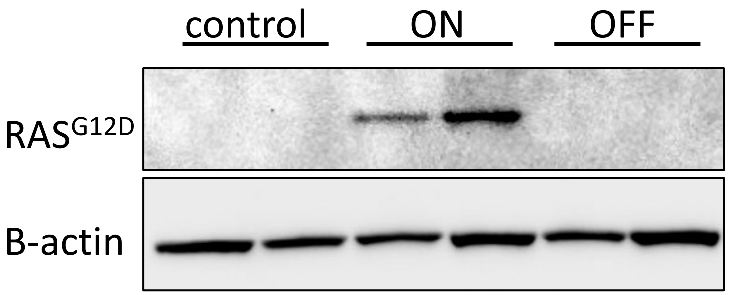

Ras (D2C1) cell signaling 8955

Raw data for Figure 1D

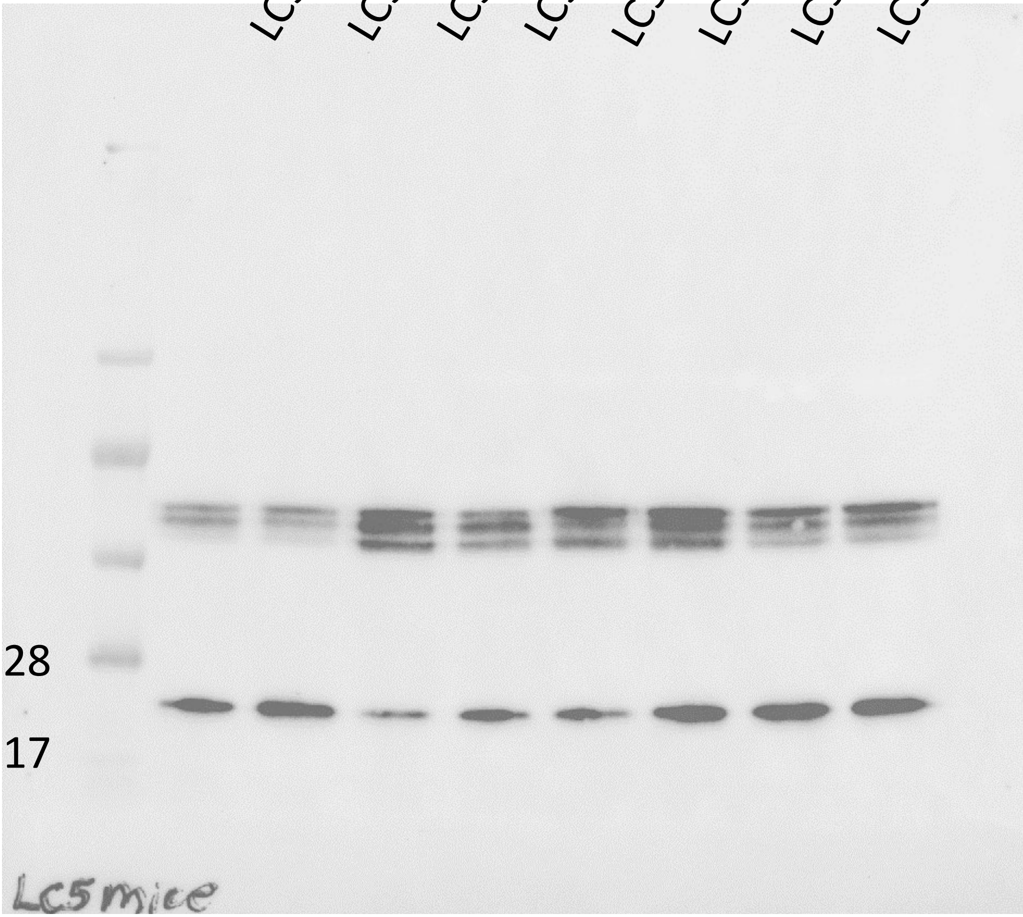

LR-#8

Control KRAS On KRAS Off KRAS On/Off/On

Raw data for Figure 1D

LC5 Lung Tissue 1023
LC5 Lung Tissue 956
LC5 Lung Tissue 817
LC5 Lung Tissue 849
LC5 Lung Tissue 1049
LC5 Lung Tissue 1015
LC5 Lung Tissue 1013
LC5 Lung Tissue 1018

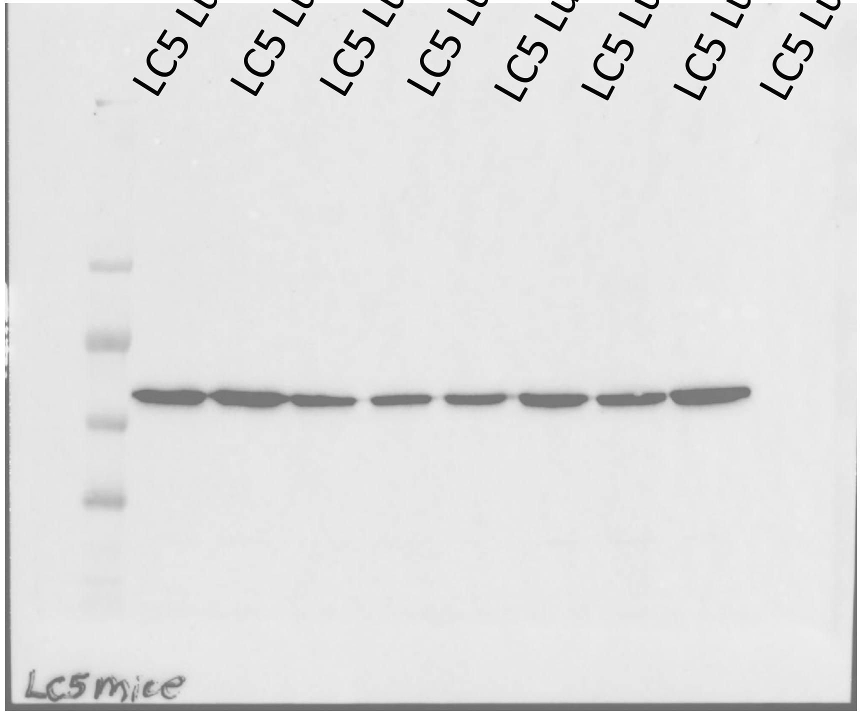

bActin HRP

LR-#8

Raw data for Figure 5B

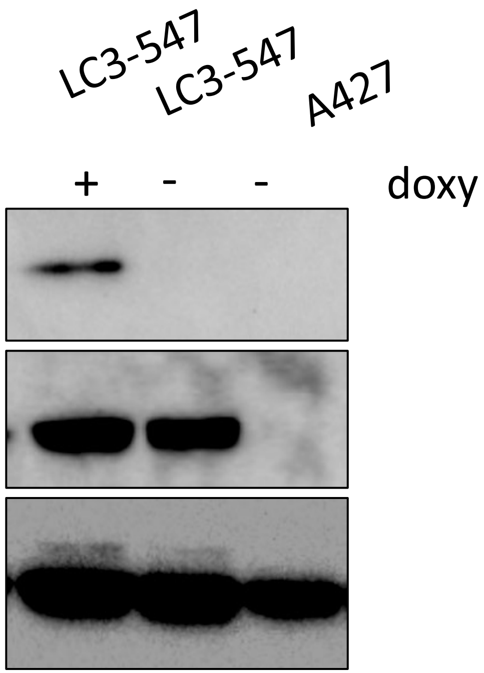

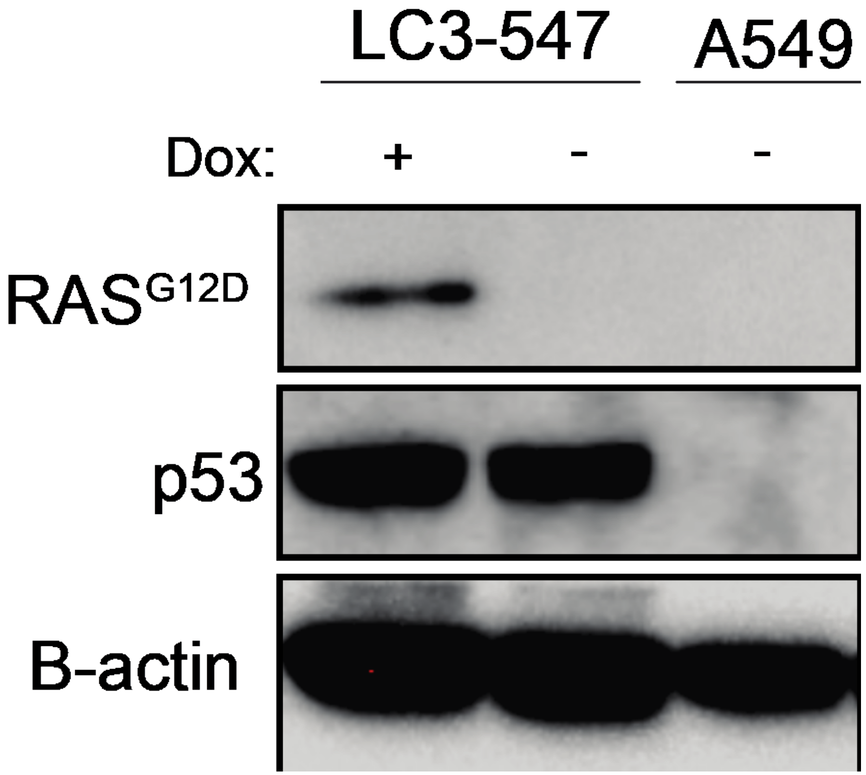

IB 113

Raw data for Figure 5B

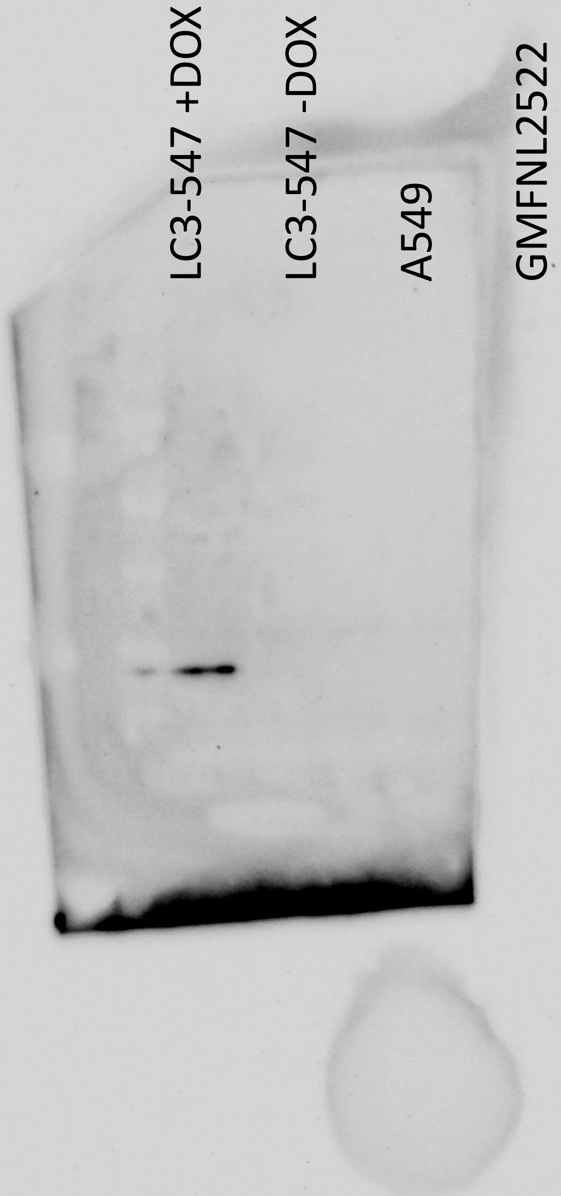

RAS^{G12D}

IB 113

Raw data for Figure 5B

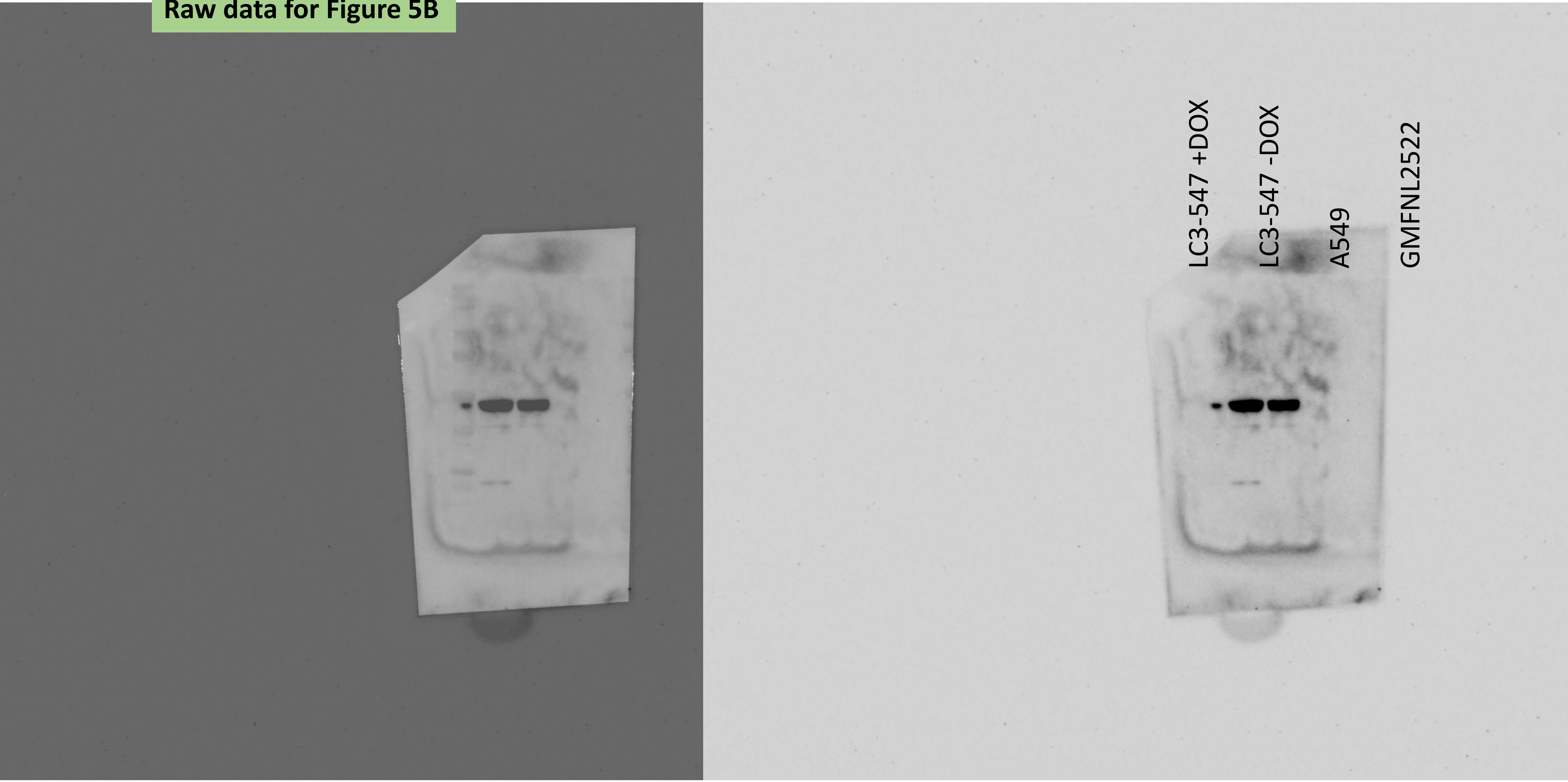

p53

Raw data for Figure 5B

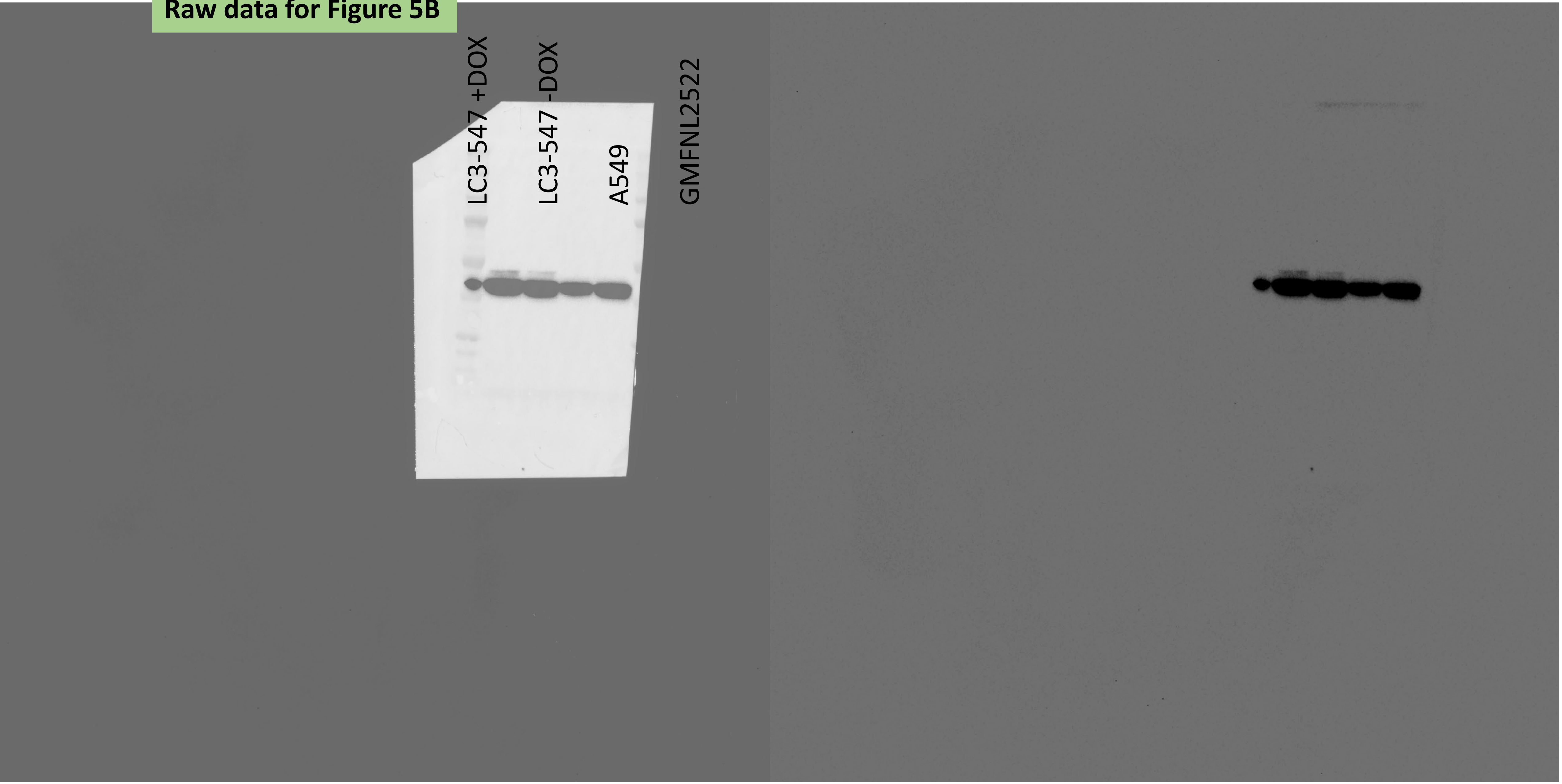

B-Actin

Raw data for Figure 5C

RH15

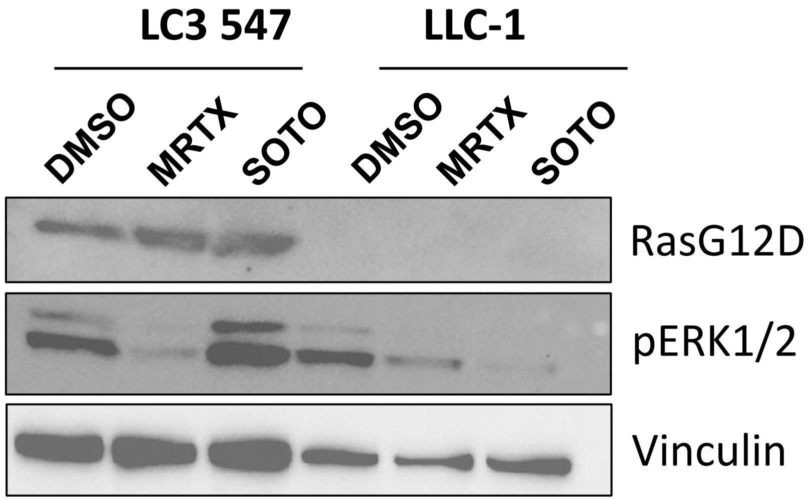

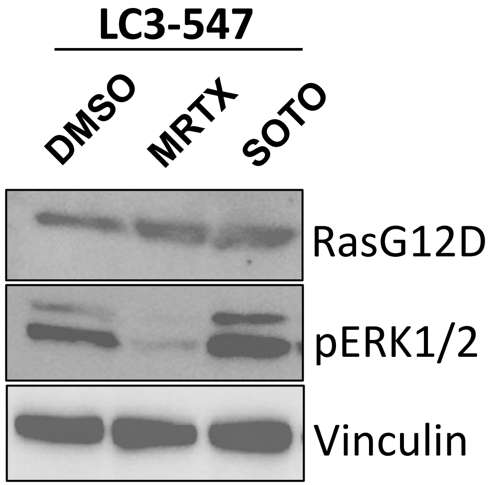

Gel #1

Raw data for Figure 5C

RH15

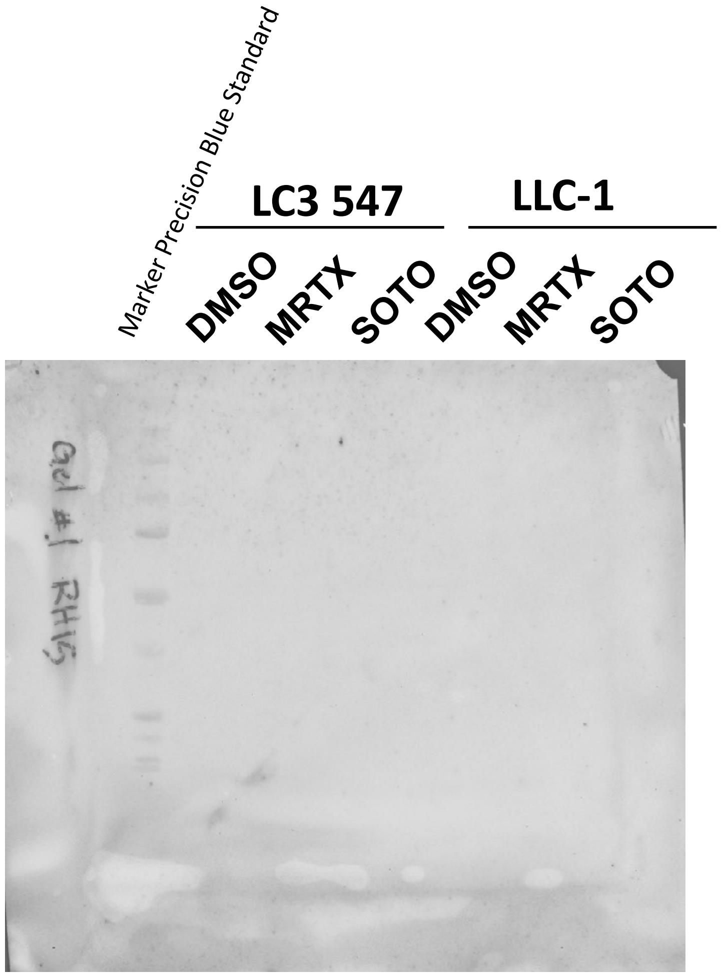

1st Antibody – RasG12D
21-25 kDa

#14429

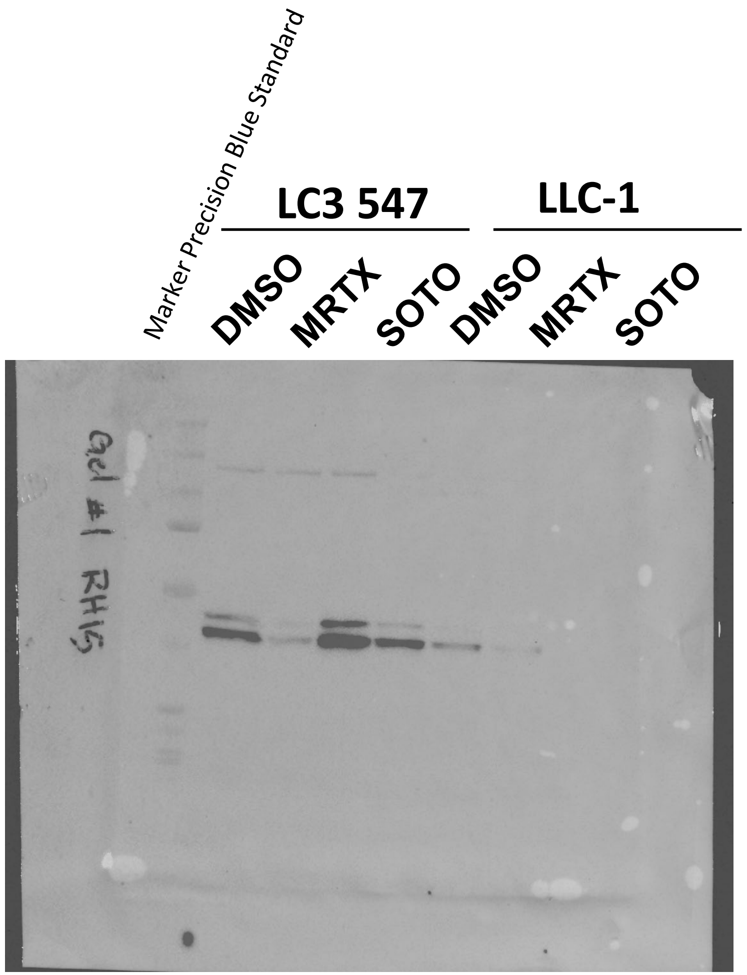

2nd Antibody – phospho-ERK
42, 44 kDa

#4370

Gel #1

Raw data for Figure 5C

RH15

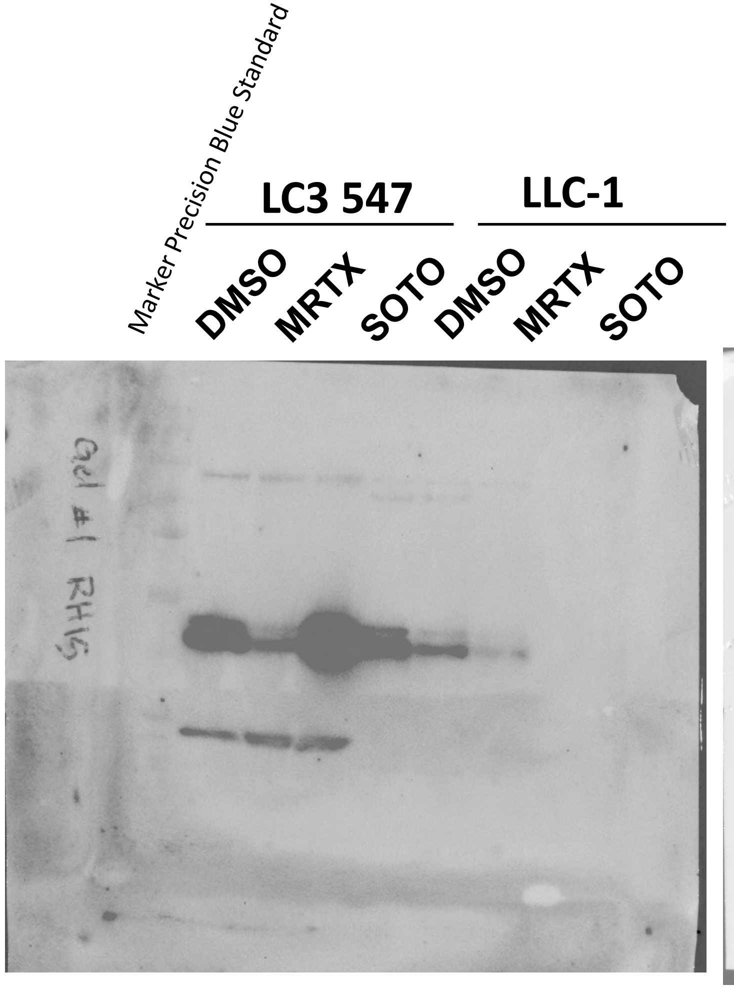

3rd Antibody – RasG12D-rerun
21-25 kDa

#14429

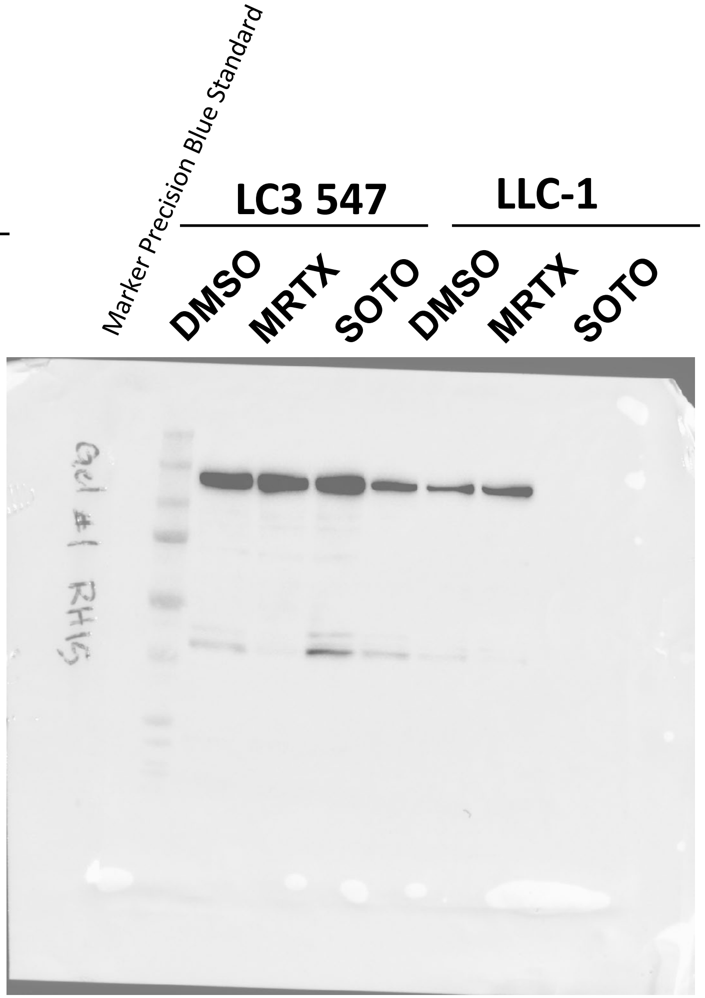

4th Antibody – Vinculin
117 kDa

#13901s

Raw data for Figure 5J

LR-# 6

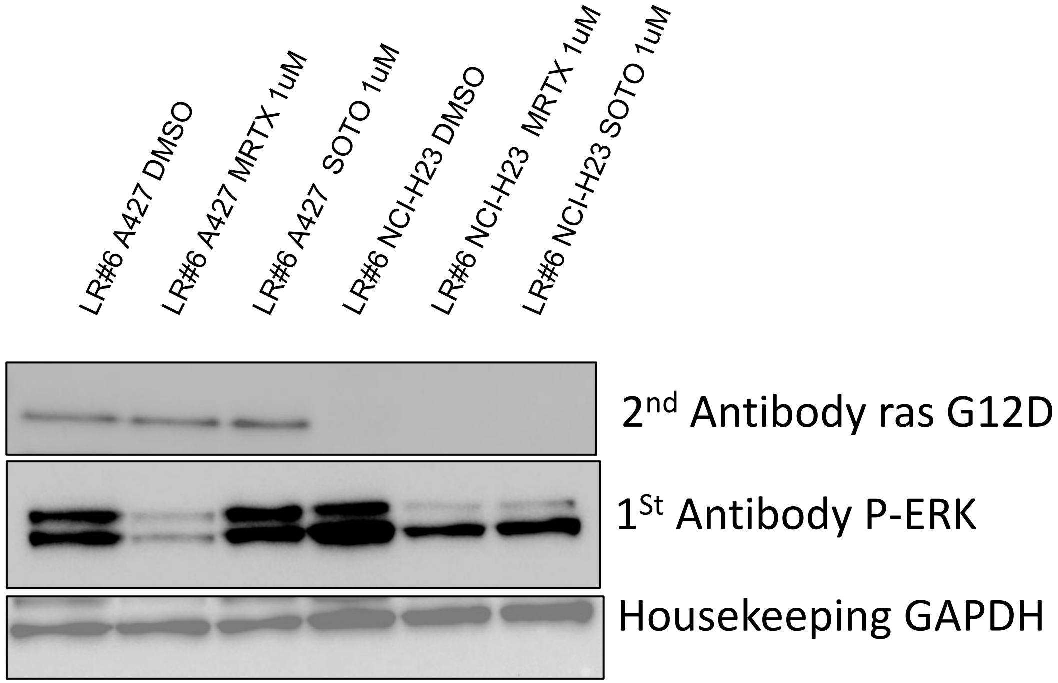

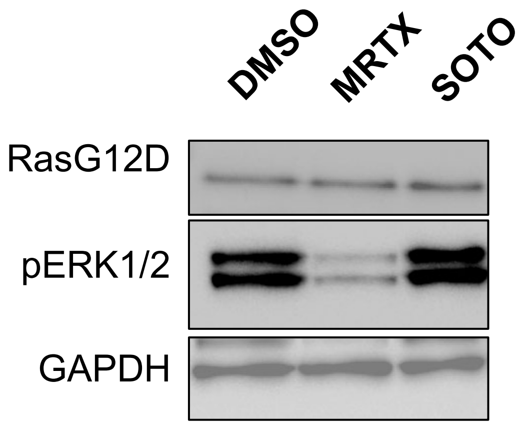

Raw data for Figure 5J

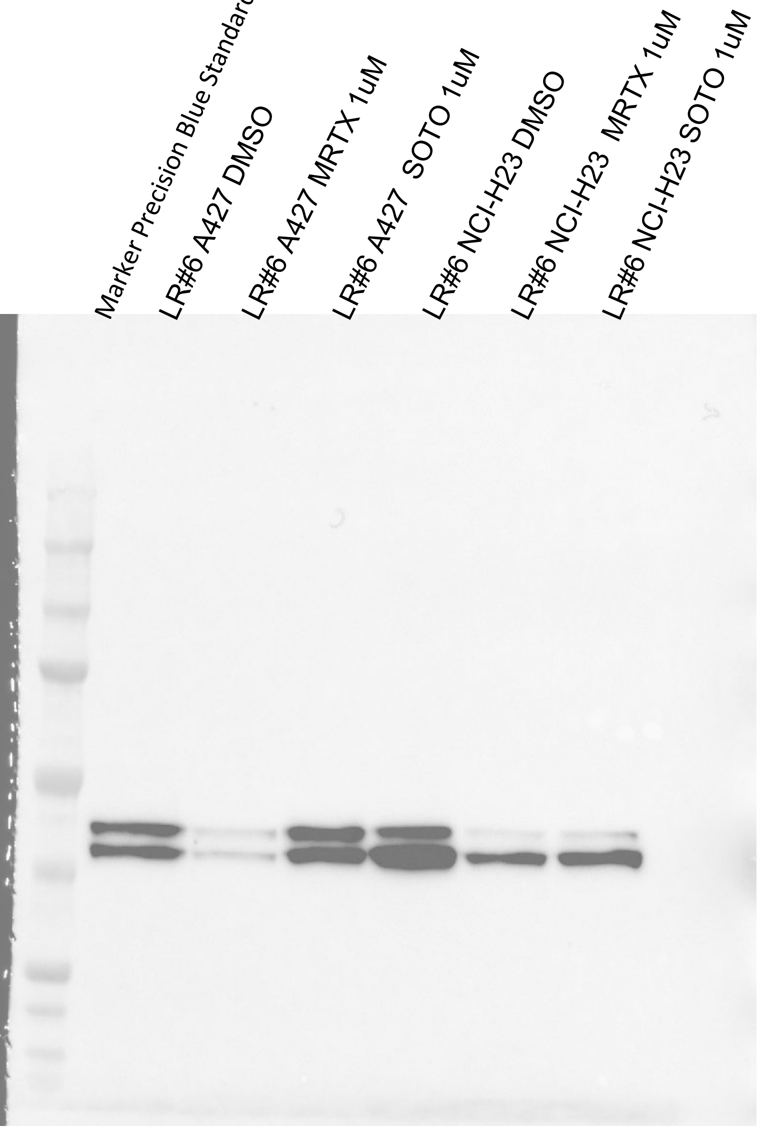

1st Antibody P-ERK

44, 42 kDa

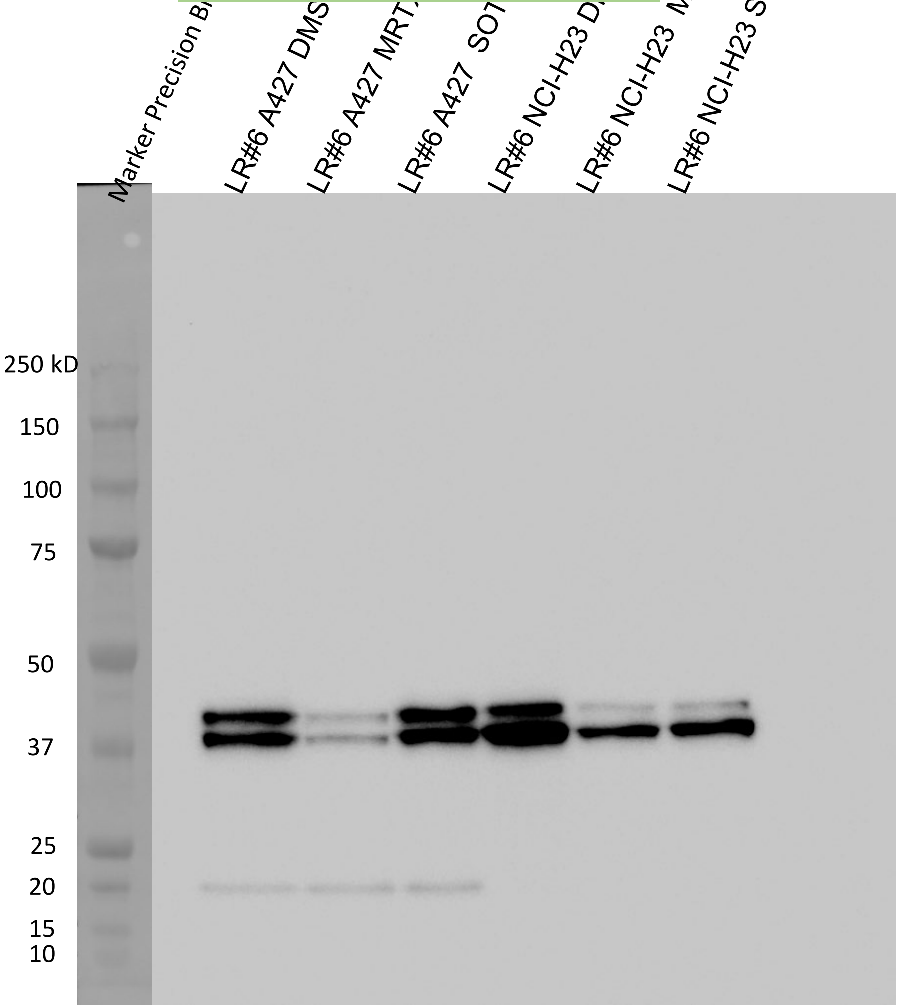

2nd Antibody ras G12D

17 kDa

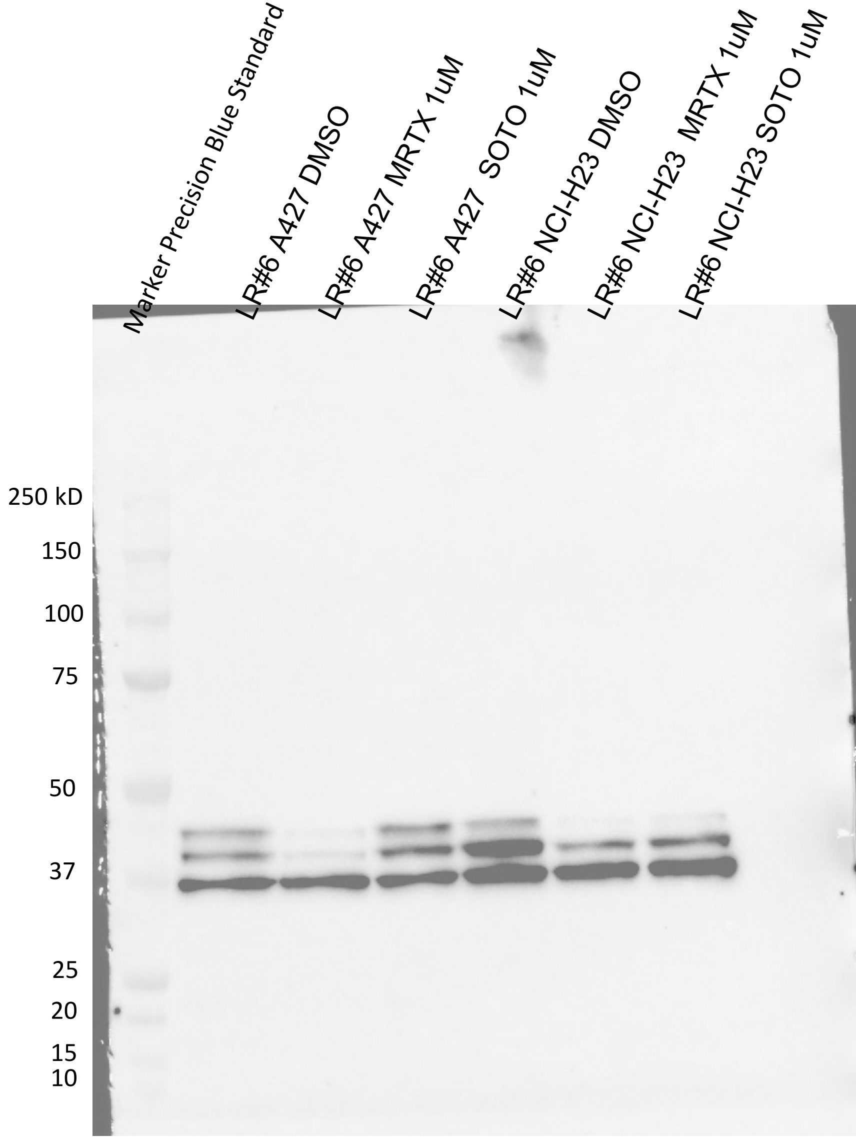

Housekeeping GAPDH

37kDa

Ran by Lily

Raw data for Figure 5J

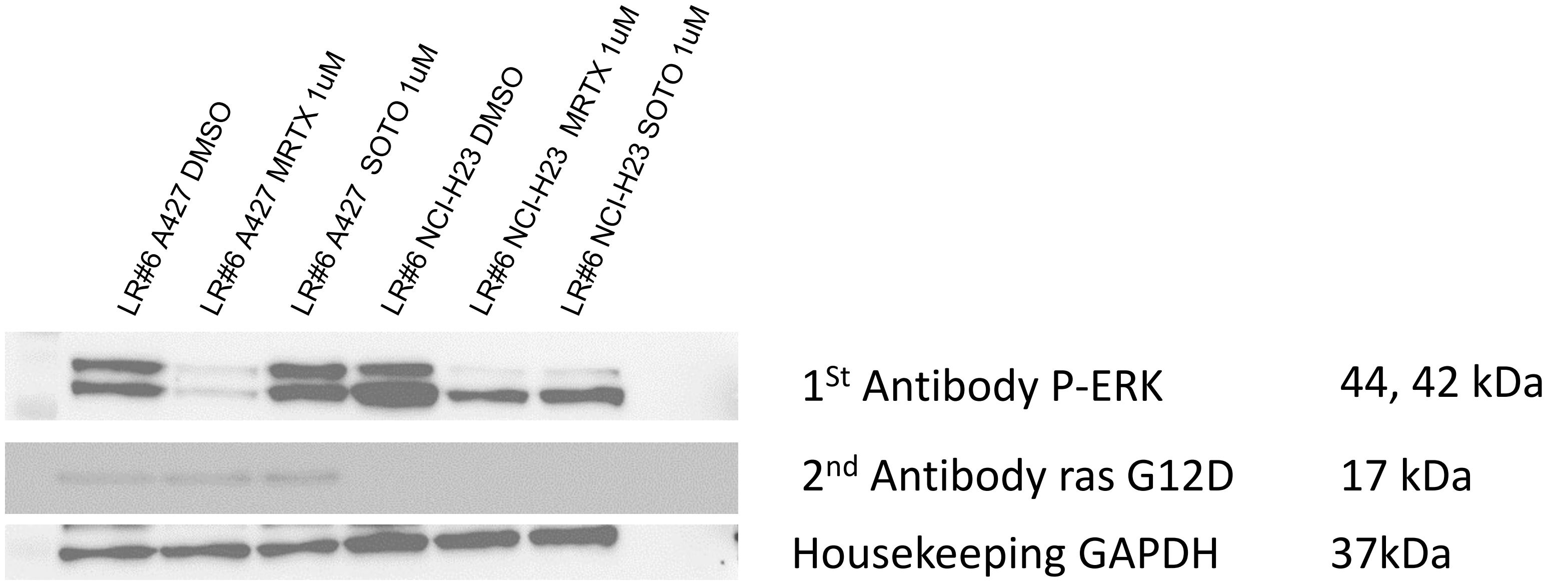

Raw data for Figure 5J

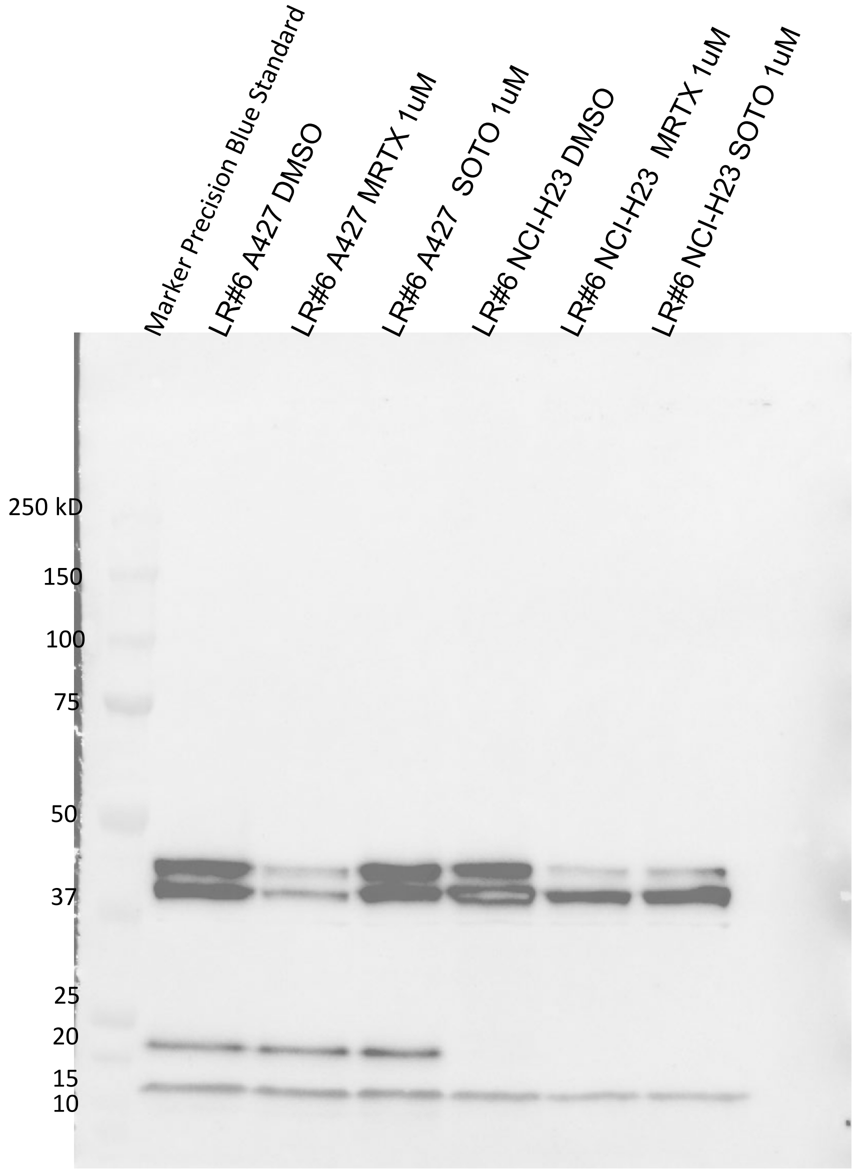

4th Antibody ras G12D Rerun

17 kDa

Used re-probe for final figure

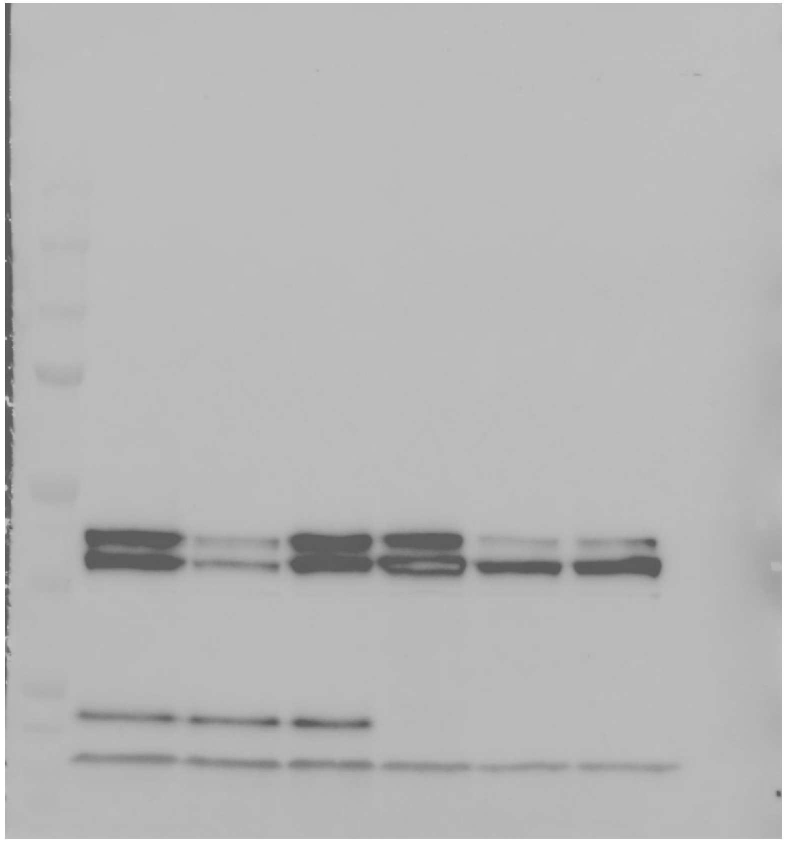

Raw data for Supplemental Figure 5D

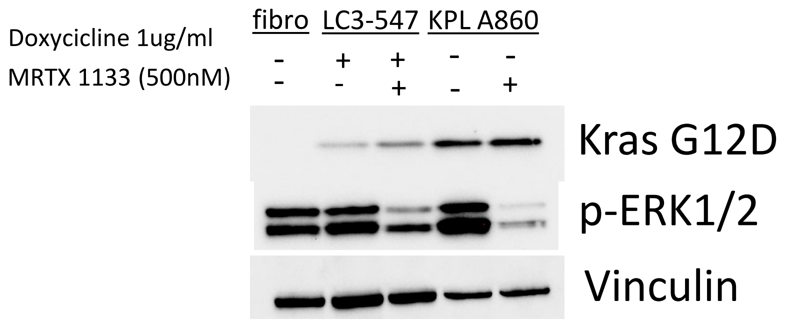

Raw data for Supplemental Figure 5D

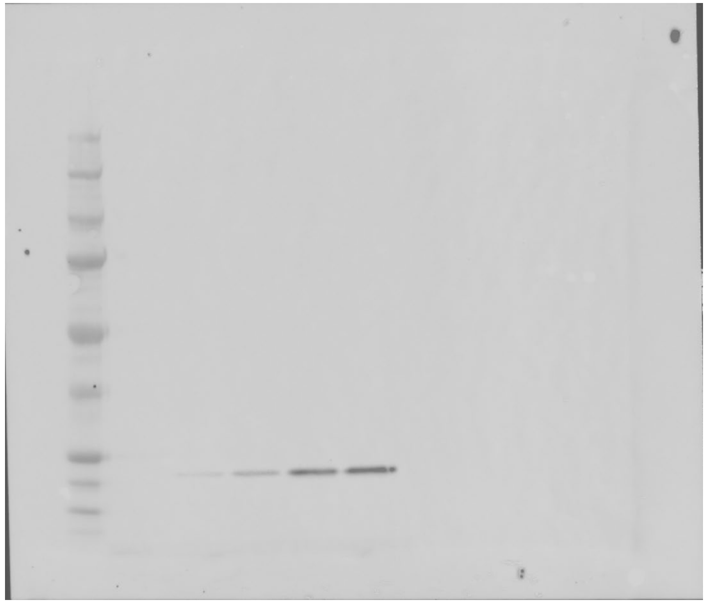

ras G12D CS# 14429

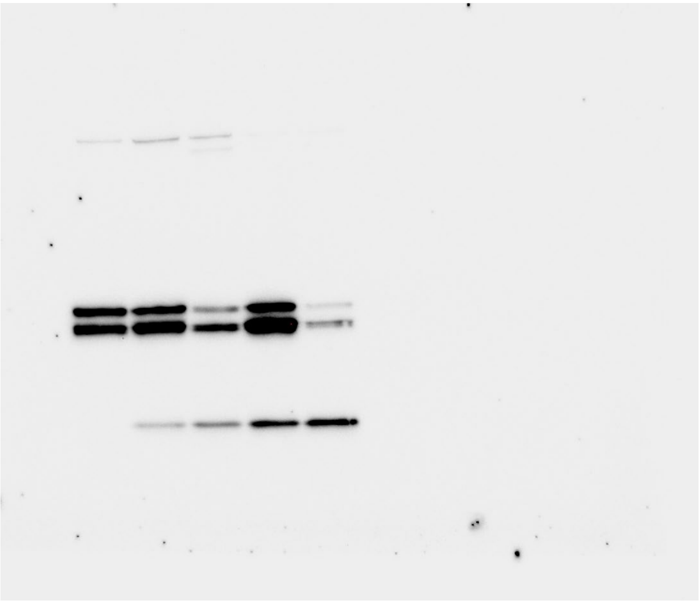

pERK CS# 4370

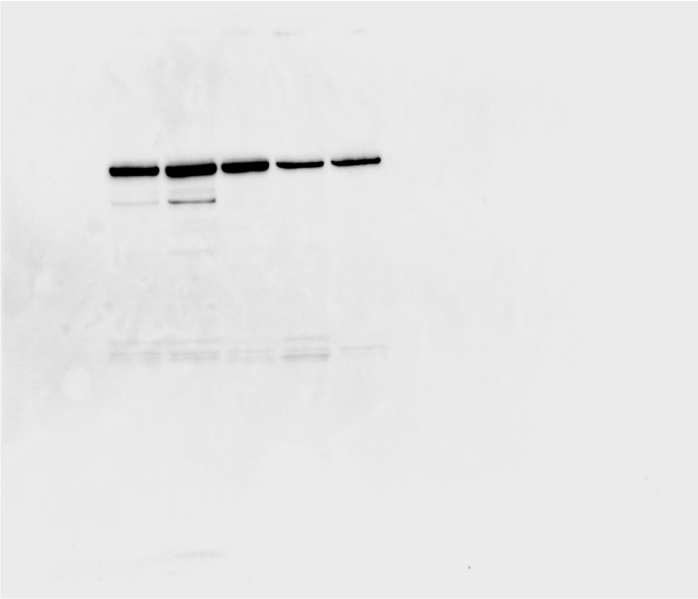

Vinculin

Raw data for Supplemental Figure 5G

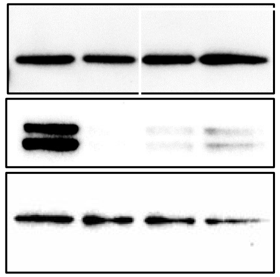

A 427 (G12D cell line) Treated with **MRTX 100 nM.**
LLC1 (G12C cell line) treated with **Sotorasib 500nM**

Raw data for Supplemental Figure 5G

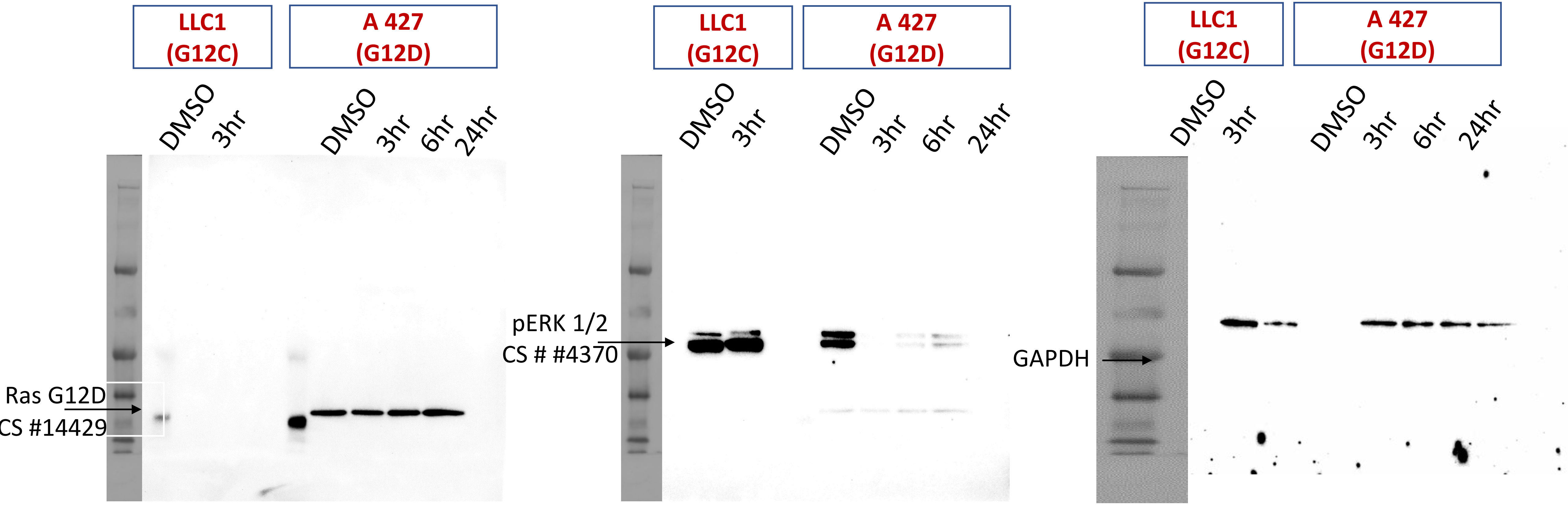

Supplement: Unedited blot and gel images [file jciinsight-10-182228-s175.pdf]
